# Method Comparison for Simulating Non-Gaussian Beams and Diffraction for Precision Interferometry

**DOI:** 10.3390/s23229024

**Published:** 2023-11-07

**Authors:** Mengyuan Zhao, Yazheng Tao, Kevin Weber, Tim Kaune, Sönke Schuster, Zhenxiang Hao, Gudrun Wanner

**Affiliations:** 1 Key Laboratory of Electronics and Information Technology for Space System, National Space Science Center, Chinese Academy of Sciences, No.1 Nanertiao, Zhongguancun, Haidian District, Beijing 100190, China; 2 University of Chinese Academy of Sciences, No.19(A) Yuquan Road, Shijingshan District, Beijing 100049, China; 3 Max Planck Institute for Gravitational Physics (Albert Einstein Institute), Callinstr. 38, 30167 Hannover, Germany; tim.haase@aei.mpg.de; 4 School of Fundamental Physics and Mathematical Sciences, Hangzhou Institute for Advanced Study, UCAS, Hangzhou 310024, Chinahaozhenxiang@ucas.ac.cn (Z.H.); 5 Institute for Gravitational Physics, Leibniz Universität Hannover, Callinstr. 38, 30167 Hannover, Germany; kevin.weber@aei.mpg.de (K.W.); soenke.schuster@aei.mpg.de (S.S.); 6 TaiJi Laboratory for Gravitational Wave Universe (Beijing/Hangzhou), University of Chinese Academy of Sciences, Beijing 100049, China

**Keywords:** optical simulation, diffraction, space interferometry

## Abstract

In the context of simulating precision laser interferometers, we use several examples to compare two wavefront decomposition methods—the Mode Expansion Method (MEM) and the Gaussian Beam Decomposition (GBD) method—for their precision and applicability. To assess the performance of these methods, we define different types of errors and study their properties. We specify how the two methods can be fairly compared and based on that, compare the quality of the MEM and GBD through several examples. Here, we test cases for which analytic results are available, i.e., non-clipped circular and general astigmatic Gaussian beams, as well as clipped circular Gaussian beams, in the near, far, and extremely far fields of millions of kilometers occurring in space-gravitational wave detectors. Additionally, we compare the methods for aberrated wavefronts and their interaction with optical components by testing reflections from differently curved mirrors. We find that both methods can generally be used for decomposing non-Gaussian beams. However, which method is more accurate depends on the optical system and simulation settings. In the given examples, the MEM more accurately describes non-clipped Gaussian beams, whereas for clipped Gaussian beams and the interaction with surfaces, the GBD is more precise.

## 1. Introduction

In classic optics textbooks, e.g., [1,2,3,4], diffraction is defined as the phenomenon that occurs when a wave is obstructed while propagating. This, for example, is the case when a Gaussian beam is clipped by an aperture. This phenomenon has been known for centuries, and there are various methods for computing diffracted wavefronts and their propagation. The most classic approach is the evaluation of diffraction integrals, such as the Fresnel–Kirchhoff diffraction formula or the Fraunhofer diffraction equation, as described in, e.g., [5].

The propagation of diffracted light closely relates to the propagation of arbitrary wavefronts for which there exists no analytic propagation equation. Such arbitrary wavefronts include all clipped and diffracting beams, as well as aberrated wavefronts. Therefore, the same methods are used for diffracted-light and aberrated wavefronts.

Even though we have known about diffraction and aberration for a long time, there seems to be no suitable method at hand that allows propagating diffracting beams with high precision through complex optical setups, where the beam repeatedly reflects and refracts at various tilted or even curved surfaces. Given our particular context of space-based interferometry for gravitational wave detection, i.e., the gravitational wave detectors LISA (Laser Interferometer Space Antenna) [6] and Taiji [7], our simulation methods are required to provide at least picometer resolution.

This precision needs to be achieved for the propagation of the light received from a remote spacecraft, i.e., a beam that is clipped by the mirrors of a telescope, followed by an optical bench carrying in the order of 50–100 components, where the beam may be clipped at various points as it propagates through the three-dimensional optical layout until it interferes with a reference beam on a photodiode [8]. Furthermore, this precision needs to be achieved for the beam transmitted toward a remote spacecraft. This entails a laser beam traveling through a three-dimensional layout, potentially with clipping causing diffraction, until it reflects from the final telescope mirror and propagates over a distance of 2.5 $\times \;10^{9}$ to 3 $\times \;10^{9}$ m (cf. for instance [8,9,10]). Ideally, both cases can be combined, resulting in optical end-to-end simulations and enabling a rich set of necessary simulations, which are currently not possible in the described complexity and are solved only through simplification. Examples include straylight simulations, particularly the simulation of the coupling of jitter into the interferometric phase noise, better known as the tilt-to-length coupling noise [11,12,13,14]. However, the method comparison described in this manuscript is applicable to a wide range of fields, and the described application in space-based gravitational wave detection is provided only as context for the requirements that motivate this comparison.

Diffraction integrals are not ideal for this type of application since they are designed for free-space propagation and need non-trivial adaptation for propagation through the described complex three-dimensional layouts. However, alternative approaches allow a comparably simple propagation of diffracting wavefronts through such setups.

These alternatives are based on a decomposition into fundamental or higher-order Hermite– or Laguerre–Gaussian beams. Once the diffracting beam is decomposed, it can be easily propagated using well-known and fast algorithms (e.g., as described in [15]). These methods involve simple ray tracing for the beam axis and the use of the ray transfer matrix formalism [4] for the propagation of the Gaussian *q*-parameter in wavefront propagation.

These decomposition methods are well established but are used under a variety of different names. The decomposition into higher-order Hermite– or Laguerre–Gaussian modes, which all share the same beam axis and beam parameters, was first proposed in [16]. Like [17,18,19], we refer to this method as the Mode Expansion Method (MEM). It is also known as modal decomposition [20] or truncated orthogonal-series expansion [21]. If Laguerre–Gaussian modes are used for the decomposition, the method is referred to as the Laguerre–Gauss expansion [22], Laguerre–Gaussian series expansion method [23], or Laguerre–Gaussian mode decomposition [24], and if Hermite–Gaussian modes are used, it is referred to as the truncated Hermite–Gauss series expansion [25].

Another decomposition method, which involves the concept of decomposing an arbitrary wavefield into Gaussian beams, was proposed by Popov in 1982 for acoustics [26]. A similar idea was conceived by Graynolds in 1981 [27], when he began developing a ray-tracing code that eventually became the commercial software ASAP (version number 1.0) and published his paper on the subject in 1985 [27,28]. In the original description, the fundamental Gaussians were all parallel, all had the same waist size, and the waist was positioned in the decomposition plane. We refer to this original version of the method proposed by Graynolds as the Gaussian Beam Decomposition (GBD) and use this terminology throughout this paper. The original method was adapted over time, for instance, with non-parallel grid beams or grid beams with an initial wavefront curvature, and implemented in several common commercial software tools, including ASAP [29], FRED, and Code V [30]. It is, unfortunately, proprietary, and it is unknown in which form or adaptation the method was implemented in the different software tools; however, this shows that the method is well established. The method of decomposing wavefronts into Gaussian beams and its adaptations are also often referred to as Gaussian beam summation [31,32], Gaussian beam superposition [33], Gaussian beamlet decomposition [30], and Gaussian beamlet summation [34], and in Code V, the method is known as Beam Synthesis Propagation (BSP) [35]. Code V’s BSP decomposes the wavefront into Gaussian beams emitted from a single point but in various directions, as opposed to decomposing the wavefront into Gaussian beams on a grid. Additionally, there are some adaptations made without changing the name. An improved GBD technique was suggested by Tanushev et al. for decomposing high-frequency wavefields into a sparse set of Gaussian beams. The selection principle used to determine the Gaussian beam parameters aims to minimize the energy difference between the original wavefield and the superimposed Gaussian beams [36]. In order to compute the scalar diffraction field of a two-dimensional field specified on a curved surface, Şahin et al. proposed an improved Gaussian Beam Decomposition (GBD) method. The three-dimensional field is expressed as a summation of Gaussian beams, each propagating in a different direction, with waist positions located at discrete points on the curved surface, obtained through regular sampling [37]. Worku et al. introduced a revised Gaussian Beam Decomposition (GBD) method that enables the computation of vectorial field propagation through high numerical aperture (NA) objectives. In their study, the decomposed Gaussian beams were polarized [38]. Worku et al. [39] presented a modified GBD that decomposes arbitrary fields with smooth wavefronts into fundamental Gaussian beams with initial curvatures. Finally, half- or quarter-Gaussian beams have been proposed for application in the GBD to optimize the simulation of sharp beam edges after passing through a hard aperture with an arbitrary shape [40].

Despite the numerous publications that utilize these methods, few publications describe and compare the MEM and GBD in detail. Therefore, the optimal settings for the methods are often unknown, and the limitations of these methods are unclear.

For the MEM, Borghi et al. [22] derived the optimal decomposition parameters using Laguerre–Gaussian (LG) modes for circular symmetric fields of a 1 mm radius, particularly for top-hat beams. Yan Rong et al. [41] extended Borghi’s optimal rule to an arbitrary radius of aperture. Liu et al. [25] presented the optimal decomposed beam waist for plane waves clipped by an arbitrary radius of the aperture with Hermite–Gaussian (HG) modes.

Regarding the GBD, the publication status seems fairly sparse. About 30 years after the proposal of the basic concept of decomposing arbitrary electric fields into fundamental Gaussian beams, in Graynolds’ overview article [27], he revisited the GBD, described the method’s history and development over time, and provided detailed implementation steps for the field decomposition, tracing, and computation of the resulting field in the target plane. In [42], various examples of modeling complex optical phenomena using the GBD were demonstrated, including interference and diffraction. However, none of these papers included a discussion of parameter settings, i.e., what waist size or waist location should be chosen for the grid beams, what overlap the grid beams should have, or what type of grid would be ideal.

As previously mentioned, recent publications have further advanced the development of the GBD method. However, again, they have neither addressed the question of parameter settings nor compared the performance of the GBD method to that of the MEM.

In this paper, we specify our experience values for parameter settings in the GBD when it is used to simulate simple cases, such as non-clipped and clipped Gaussian beams, for which we have analytical results available for comparison. We then directly compare the performance of the MEM and the original GBD method as introduced by Graynolds. This comparison is performed for propagation distances that vary significantly, ranging from the common case of a few millimeters in the very near field to millions of kilometers. With this exceptionally large propagation distance, we test the applicability of the methods in the context of space interferometry, particularly space-gravitational wave detectors like LISA and Taiji, for which the properties of the electric field need to be characterized for distances up to 3 million kilometers. Additionally, we qualitatively test the MEM and GBD in decomposing and propagating aberrated wavefronts, for which we do not have an analytic result for comparison. Finally, we test the propagation through an optical setup by reflecting the decomposed fields from curved mirrors with different curvatures.

Following the introduction, we summarize the properties of the MEM and GBD in Section 2, define the errors for judging deviations of the decomposed representation of the beam from the exact one, and test the methods individually using analytically known cases. In Section 3, we prepare the direct comparison of both methods, first by defining what we deem to be a fair comparison and then by testing which settings of the methods result in such a fair comparison. In Section 4, we perform the direct comparison of the two methods using various test cases, i.e., for non-clipped and clipped Gaussian beams, aberrated wavefronts, and the reflection of a Gaussian beam from a spherical mirror. Finally, we provide a summary and our conclusions in Section 5.

## 2. Wavefront Decomposition Methods

In this section, we introduce the MEM and GBD in detail and individually test their performance using a simple exemplary case.

### 2.1. Properties and Individual Test of the Mode Expansion Method

#### 2.1.1. MEM: Method Description

The MEM is a well-known method, defined, for instance, in [4], which describes the decomposition of an arbitrary wavefront into higher-order Laguerre–Gaussian (LG) modes or Hermite–Gaussian (HG) modes. LG modes are radially symmetric and are, therefore, defined in cylindrical coordinates, whereas HG modes are defined in rectangular coordinates due to their axial symmetry. A conversion between both types of modes is known and described in detail, for instance, in [43,44]. Throughout this paper, we focus on decompositions using HG modes, which are defined as

(1) $$\begin{array}{c} \begin{array}{cc} {\mathrm{HG}}_{mn}\left(x,y,z;w_{0d}\right) & =\frac{c_{mn}}{w(z)}H_{m}\left(\frac{\sqrt{2}x}{w(z)}\right)H_{n}\left(\frac{\sqrt{2}y}{w(z)}\right)\\ \; & \;\cdot \;\exp\left(-\frac{x^{2}+y^{2}}{w^{2}\left(z\right)}\right)\exp\left(-ik\frac{x^{2}+y^{2}}{2R(z)}+i\left(m+n+1\right)\zeta \left(z\right)\right)\exp\left(-ikz\right), \end{array} \end{array}$$

where $w_{0d}$ is the waist of the fundamental mode HG${}_{00}$, which is used as a parameter for all HG modes, and the beam radius w(z), the radius of the curvature R(z), and the Gouy phase ζ(z) have the same definitions as for fundamental Gaussian beams. The coefficients $c_{mn}$ are normalization constants:

(2) $$c_{mn}=\sqrt{\frac{2}{\pi }}\frac{1}{\sqrt{m!2^{m}}}\frac{1}{\sqrt{n!2^{n}}}\;,$$

where the function $H_{m}\left(•\right)$ is the $m^{\mathrm{th}}$ Hermite polynomial, provided in [4],

(3) $$H_{m}\left(x\right)={\left(-1\right)}^{m}e^{x^{2}}\frac{d^{m}}{dx^{m}}e^{-x^{2}}\;.$$

An important property of these HG modes is that they are orthonormal and complete and, therefore, form a basis [4]:

(4) $$\;\int \int \;{\mathrm{HG}}_{mn}^{*}\left(x,y,z;w_{0d}\right){\mathrm{HG}}_{kl}\left(x,y,z;w_{0d}\right)\;dxdy=\delta_{mk}\delta_{nl}\;,$$

where HG${}_{mn}^{*}\left(x,y;w_{0d}\right)$ is the complex conjugated Hermite–Gaussian mode, and $w_{0d}$ is the waist of the fundamental mode HG${}_{00}$, which is used as a parameter for all HG modes. The Kronecker delta function $\delta_{mk}$ equals 1 for m=k and equals 0 if m≠k. This implies that any wavefront E(x,y) can be decomposed into a superposition $E_{\infty }\left(x,y\right)$ of these modes [4]:

(5) $$E_{\infty }\left(x,y\right):=\sum_{m=0}^{+\infty }\sum_{n=0}^{+\infty }a_{mn}{\mathrm{HG}}_{mn}\left(x,y;w_{0d}\right)\;,$$

where $E_{\infty }\left(x,y\right)$ is a mathematically exact representation of E(x,y):

(6) $$E_{\infty }\left(x,y\right)\equiv E\left(x,y\right)\;,$$

where ≡ indicates that the functions are equivalent for every point (x,y). The complex coefficients $a_{mn}$ with $|a_{mn}{|}^{2}=P_{mn}$ are usually referred to as mode overlap and describe how much beam power $P_{mn}$ is stored in each mode. They can be calculated using the inner product [4]

(7) $$a_{mn}=\;\int \int \;{\mathrm{HG}}_{mn}^{*}\left(x,y;w_{0d}\right)E\left(x,y\right)dxdy\;.$$

In real computations, it is not possible to use either an infinite number of modes in the decomposition (Equation (5)) or an infinite overlap integral (Equation (7)) to determine the mode overlap. Replacing the infinite surface integral in Equation (7) with a finite one is uncritical, provided that the surface is chosen to be sufficiently large because electric fields of interest usually fade out toward higher radial distances. Therefore, by choosing appropriately large integration boundaries, the introduced error becomes negligible. However, the error made by working with a finite mode order *N* is often non-negligible. Consequently, the decomposed field is no longer an exact representation of the input field E(x,y) and is only an approximation:

(8) $$E\left(x,y\right)\approx E^{N}\left(x,y,w_{0d}\right)=\sum_{m=0}^{N}\sum_{n=0}^{N-m}a_{mn}{\mathrm{HG}}_{mn}\left(x,y;w_{0d}\right)\;.\;$$

Here, we refer to *N* as the maximum mode order of the MEM. In Equation (8), we use a triangular summation of the modes by summing *n* only up to N−m rather than *N* [44]. This ensures that within any decomposition, all polynomials up to the given order *N* are considered and no polynomial orders larger than *N* are included. Consequently, in any MEM with mode order *N*, there are (N+1)(N+2)/2 HG modes superimposed. For radially symmetric fields E(x,y), the overlap $a_{mn}$ is set to zero if either the index *m* or *n* is odd. This means that for any mode order *N*, the number ν of modes used in the MEM is given by

(9) $$\nu =\left\{\begin{array}{cc} (N+1)(N+2)/2 & \mathrm{if}\;\mathrm{the}\;E(x,y)\;\mathrm{is}\;\mathrm{non}-\mathrm{symmetric}\;\\ (\lfloor N/2\rfloor +1)(\lfloor N/2\rfloor +2)/2 & \mathrm{if}\;\mathrm{the}\;E(x,y)\;\mathrm{is}\;\mathrm{symmetric}. \end{array}\right.$$

where the notation ⌊•⌋ represents the floor of the value.

#### 2.1.2. Error Definitions for the MEM

The finite mode order decomposition provided in Equation (8) is not exact and will, therefore, have an error. It can be learned that for any given mode order, the decomposition error of the MEM depends on the mode order and the waist size $w_{0d}$ chosen for the modes in the decomposition. Using the norm

(10) $${∥f(x,y)∥}^{2}={\;\int \int \;}_{R^{2}}{\left|f(x,y)\right|}^{2}dxdy\;,$$

the *normalized mean-squared error (NMSE)* is given by

(11) $$\begin{array}{cc} \varepsilon^{\mathrm{NMSE}}\left(N,w_{0d}\right) & :=\frac{{∥E^{N}\left(x,y,w_{0d}\right)-E\left(x,y\right)∥}^{2}}{{∥E(x,y)∥}^{2}}\\ \; & =\frac{{∥E(x,y)∥}^{2}}{{∥E(x,y)∥}^{2}}-2ℜ\frac{E^{N}\left(x,y,w_{0d}\right)E^{*}\left(x,y\right)}{{∥E(x,y)∥}^{2}}+\frac{{∥E^{N}\left(x,y,w_{0d}\right)∥}^{2}}{{∥E(x,y)∥}^{2}}\\ \; & =1-2ℜ\left(\sum_{m=0}^{N}\sum_{n=0}^{N-m}a_{mn}{\;\int \int \;}_{R^{2}}E^{*}\left(x,y\right){\mathrm{HG}}_{mn}\left(x,y;w_{0d}\right)dxdy\right)/{∥E(x,y)∥}^{2}\\ \; & +\left(\sum_{m=0}^{N}\sum_{n=0}^{N-m}a_{mn}^{2}{\;\int \int \;}_{R^{2}}{{\mathrm{HG}}^{2}}_{mn}\left(x,y;w_{0d}\right)dxdy\right)/{∥E(x,y)∥}^{2} \end{array}$$

(12) $$\begin{array}{cc} \; & =1-\frac{\sum_{m=0}^{N}\sum_{n=0}^{N-m}a_{mn}^{2}}{P}\;, \end{array}$$

using ${∥E(x,y)∥}^{2}=P$, where *P* is the power of the initial beam. For any input field E(x,y), this normalized mean-squared error $\varepsilon^{\mathrm{NMSE}}\left(N,w_{0d}\right)$ depends solely on the mode order *N* and waist size $w_{0d}$ chosen during the decomposition, and it has the property of being propagation distance-independent (cf. Equation (7), and [22]).

The NMSE is defined via infinite surface integrals, which are replaced by numerical integrals over finite surfaces in optical simulations. This means that in simulations, a *discretized NMSE (DNMSE)* $\varepsilon_{o}^{\mathrm{DNMSE}}\left(N_{R},R,z\right)$ is evaluated. For radial surfaces and assuming radially symmetric beams, this is given by

(13) $$\begin{array}{cc} \varepsilon_{\circ }^{\mathrm{DNMSE}}\left(N_{R},R,z\right) & :=\frac{\sum_{i=0}^{N_{R}}2\pi {\left|E^{N}\left(r_{i},z,w_{0d}\right)-E\left(r_{i},z\right)\right|}^{2}r_{i}\Delta r}{{∥E(x,y,z)∥}^{2}}\\ \; & =\frac{\sum_{i=0}^{N_{R}}2\pi {\left|E^{N}\left(r_{i},z,w_{0d}\right)-E\left(r_{i},z\right)\right|}^{2}r_{i}\Delta r}{P}\;, \end{array}$$

and for non-radially symmetric beams on rectangular surfaces, it is given by

(14) $$\varepsilon_{□}^{\mathrm{DNMSE}}\left(N_{X},N_{Y},X,Y,z\right):=\sum_{i=1}^{N_{X}}\sum_{j=1}^{N_{Y}}\frac{{\left|E^{N}\left(x_{i},y_{j},z,w_{0d}\right)-E\left(x_{i},y_{j},z\right)\right|}^{2}\Delta x\Delta y}{P}\;.$$

Here, $r=\sqrt{x^{2}+y^{2}}$ denotes the radial distance; $N_{R},N_{X},$ and $N_{Y}$ are the numbers of sampling points; and Δr, Δx, and Δy are the step sizes in the different directions, such that the maximal distances are $R=N_{R}\Delta r$, $X=N_{X}\Delta X$, and $Y=N_{Y}\Delta Y$. Due to the assumed radial symmetric beams, we substituted ${\;\int \int \;}_{R^{2}}dxdy$ with ∑2πrΔr in Equation (13). Only the numerator is discretized in the DNMSE because the NMSE is normalized by the initial beam’s total power *P*, which is usually known.

The discretized NMSE is a numerical representation of the NMSE; therefore, the propagation distance is independent, provided that enough sampling points are chosen and the radial distance *R* is sufficiently large. However, this implies that with non-ideal settings, such as too few sampling points or too small a radial range, the error is indeed propagation distance-dependent, which we highlight in Equation (13) by the explicitly stated *z*-dependency.

One disadvantage of all the shown errors is that they provide only integrated information and no distribution over a plane. We, therefore, define a sampling point-dependent error $\varepsilon^{\mathrm{rel}}\left(x_{i},y_{i},z\right)$, which we name the *relative error:*

(15) $$\varepsilon^{\mathrm{rel}}\left(x_{i},y_{i},z\right):=\frac{\left|E^{N}\left(x_{i},y_{i},z,w_{0d}\right)-E\left(x_{i},y_{i},z\right)\right|}{\left|E(x_{i},y_{i},z)\right|}\;.$$

For radially symmetric beams, we sample along the *x*-axis by setting y=0. Therefore, the reduced 1D version of Equation (15) can be written as:

(16) $$\varepsilon^{\mathrm{rel}}\left(r_{i},z\right)=\varepsilon^{\mathrm{rel}}\left(x_{i},0,z\right)=\frac{\left|E^{N}\left(x_{i},0,z,w_{0d}\right)-E\left(x_{i},0,z\right)\right|}{\left|E(x_{i},0,z)\right|}\;.$$

Throughout this paper, we use these relative errors to visualize the performance of the MEM, as well as for a qualitative comparison of the MEM and GBD. To quantify the resulting information and assess the total error in the finite surface of interest, we define the summed relative error, for rectangular target surfaces and no assumed symmetry, or circular symmetric beams on a circular surface as

$$\begin{array}{cc} \varepsilon_{\sum }^{\mathrm{rel}}\left(N_{X},N_{Y},X,Y,z\right) & =\sum_{i=1}^{N_{X}}\sum_{j=1}^{N_{Y}}\frac{\left|E^{N}\left(x_{i},y_{j},z,w_{0d}\right)-E\left(x_{i},y_{j},z\right)\right|}{\left|E(x_{i},y_{j},z)\right|}\Delta x\Delta y \end{array}$$

(17) $$\begin{array}{cc} \; & =\sum_{i=1}^{N_{X}}\sum_{j=1}^{N_{Y}}\varepsilon^{\mathrm{rel}}\left(x_{i},y_{j},z\right)\Delta x\Delta y\;, \end{array}$$

(18) $$\begin{array}{cc} \varepsilon_{\sum }^{\mathrm{rel}}\left(N_{R},R,z\right) & :=\sum_{i=0}^{N_{R}}\frac{2\pi \left|E^{N}\left(r_{i},z,w_{0d}\right)-E\left(r_{i},z\right)\right|r_{i}\Delta r}{\left|E(r_{i},z)\right|}\;. \end{array}$$

This summed error definition is, for typical settings in optical simulations, fairly independent of the chosen number of sampling points, since Equations (17) and (18) are representations of a discretized integral and, therefore, represent the surface under the given function. However, the summed relative error is, unfortunately, not normalized.

This error condenses the findings of the relative error through a simple sum and allows a quick comparison of different simulation settings for the same setup. However, this summed relative error is only a useful measure in regions where the electric field does not vanish. Therefore, it should be interpreted and used with care. We use all introduced error types throughout this paper to study the performance of the MEM and compare it to the GBD.

#### 2.1.3. MEM Settings

When a wavefront is decomposed using the MEM, there are only two parameters that need to be chosen: the waist $w_{0d}$ of the modes used in the decomposition and the maximum mode order *N*. For any maximum mode order, the choice of $w_{0d}$ directly affects the magnitude of the mode overlap $a_{mn}$ and hence the resulting error $\varepsilon^{\mathrm{NMSE}}\left(N,w_{0d}\right)$. One can then, for instance, choose the decomposition waist $w_{0d}$ such that the mode overlap of a specific mode is maximal (e.g., as used in [19]) or the error made in the decomposition is minimal. Throughout this paper, we use the latter criterion, minimizing the error $\varepsilon^{\mathrm{NMSE}}\left(N,w_{0d}\right)$ by following the examples of [22,25,41].

Which waist is optimal for the decomposition depends on the properties of the initial wavefront E(x,y,z). Therefore, for an arbitrary wavefront, the optimal decomposition waist is unknown. However, for the common special case of circular symmetric wavefronts originating from clipping at an aperture of radius $R_{a}$, the optimal waist was found to be [22,25,41,45]:

(19) $$w_{0d}=R_{a}\sqrt{\frac{2}{N}}\;.$$

This choice results in the minimum NMSE $\varepsilon^{\mathrm{NMSE}}\left(N,w_{0d}\right)$ in Equation (12) for a given mode order *N* [22,25,41,45].

#### 2.1.4. Example: MEM Performance for a Clipped Gaussian Beam

We now demonstrate the performance of the MEM using an example, for which the electric field is analytically known. It should be noted that the examples here meet the assumptions of the paraxial approximation, where the Gaussian beam waist is significantly larger than the wavelength, so the analytical methods are considered reliable and are used as references. In this example, we investigate a clipped Gaussian beam. We assume that a Gaussian beam impinges orthogonally and is perfectly aligned to a circular aperture with a radius of $R_{a}=0.5\mathrm{mm}$. The waist of the incident Gaussian beam has a radius of $w_{0}=2\mathrm{mm}$, which is located in the aperture plane. The resulting circular symmetric clipped Gaussian beam is decomposed using the MEM with varying mode orders: N=10,20,…50. For every mode order, the waist size $w_{0d}$ of the modes is calculated using Equation (19). We compute the electric field’s amplitude and phase at various propagation distances and compare the results with the numerical evaluation of an analytic formula developed by Campbell in [46] for clipped Gaussian beams in the Fresnel region. Analogously, we use the analytical method of Tanaka et al. (cf. Equations (1)–(6) in [47]) for the Fraunhofer region. The distinction between the Fresnel and Fraunhofer regions is determined using the Fresnel number *F*, given by

(20) $$F:=\frac{R_{a}^{2}}{\lambda d}\;,$$

where λ denotes the wavelength of the beam, *d* is the propagation distance after the clipping aperture, and $R_{a}$ is the radius of the aperture. The near field refers to propagation distances that make *F* larger than 1. If the Fresnel number is smaller than 1, the beam has propagated to the far field. In this example, we use propagation distances *d* of 5mm,20mm, and 100mm, i.e., *F* is 46.9925,11.7481, and 2.3496 in the near field, and d=1000mm with F=0.2350 in the far field. The number of sampling points for requesting the complex electric field is set to 3001. For convenience, all parameters in this example are listed in Table 1.

**Figure 1:**
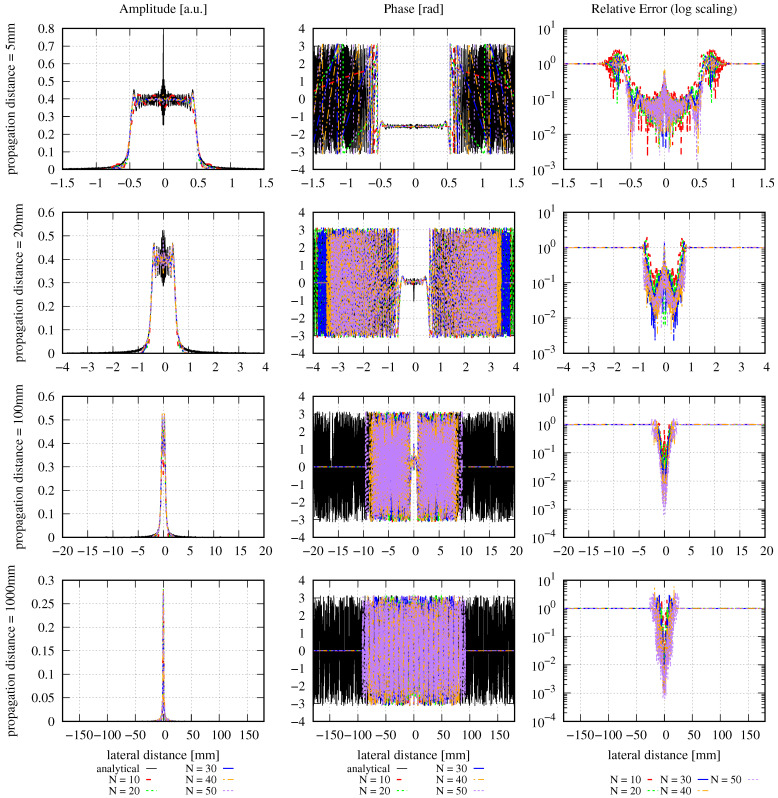
Performance of the MEM with different maximum mode orders of *N* = 10, 20, 30, 40, and 50 for an incoming circular Gaussian beam with a 2 mm waist being clipped by a 0.5 mm radius circular aperture. The amplitude (absolute value), phase, and relative error $\varepsilon^{\mathrm{rel}}\left(N_{R},R,z\right)$ are shown at different propagation distances of z= 5 mm, 20 mm, and 100 mm (near field) and 1000 mm (far field) after the clipping aperture. The analytical methods used for the near and far fields were those of Campbell [46] and Tanaka et al. [47], respectively. The lateral distances for each propagation distance are chosen to be large enough to cover all the power. For these large lateral ranges, the MEM is effectively failing, generating zero amplitudes and phases from lateral ranges that are about 3 times the spot size of the highest mode in the decomposition.

**Figure 2:**
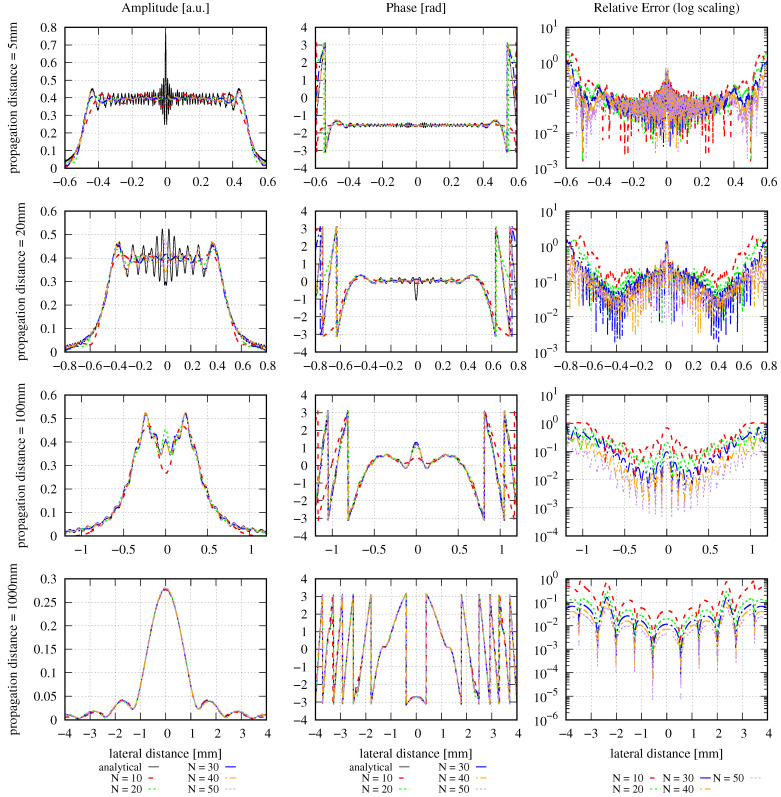
MEM performance for the same test case shown in Figure 1 but for smaller lateral ranges. The performance of the MEM with different maximum mode orders of *N* = 10, 20, 30, 40, and 50 is shown for an incoming circular Gaussian beam with a 2 mm waist being clipped by a 0.5 mm radius circular aperture. The amplitude (absolute value), phase, and relative error $\varepsilon^{\mathrm{rel}}\left(N_{R},R,z\right)$ are shown at different propagation distances of z= 5 mm, 20 mm, and 100 mm (near field) and 1000 mm (far field) after the clipping aperture. The analytical methods used for the near and far fields were those of Campbell [46] and Tanaka et al. [47], respectively. One can see that the further the beam propagates or the higher the mode order, the better the performance of the MEM.

The amplitude, phase, and relative error distributions calculated using Equation (16) are shown in Figure 1 for different propagation distances after the clipping aperture with different mode orders. The lateral ranges chosen for this figure are unusually large. The corresponding results for a smaller lateral range are shown in Figure 2. All of the introduced error types have been calculated for both choices of lateral ranges and are listed in Table 2.

The large lateral ranges used in Figure 1 were chosen so that they were large enough to make the DNMSE propagation distance-independent. In this case, the deviation between the input beam power and the MEM beam power was less than 2%. The incident beam power *P* is calculated simply from the Gaussian beam power passing through the aperture radius $R_{a}$:

(21) $$P=P_{0}\left[1-\exp\left(-2R_{a}^{2}/w_{0}^{2}\right)\right]\;,$$

with $R_{a}$ = 0.5 mm, $w_{0}$ = 2 mm, and $P_{0}$ being the full power of the Gaussian beam prior to clipping. The resulting normalized power ($P/P_{0}$) of the clipped Gaussian beam is 0.12. The power of the MEM beams were computed by the numerical sum $\sum_{i=0}^{R}2\pi {\left|E(r_{i})\right|}^{2}r_{i}\Delta r$, which is a numerical representation of the denominator of Equation (13). This procedure resulted in a slight variation of the MEM beam power at the different propagation distances. The deviations between the input beam power and the MEM beam power were 1.16%, 1.16%, 1.17%, and 1.66% for propagation distances of 5 mm, 20 mm, 100 mm, and 1000 mm, respectively. Ideally, the lateral range would be chosen from the spot size of the clipped beam at the various propagation distances. However, particularly for clipped and diffracted beams, there is not one uniquely defined spot size, but rather a number of different concurring options, which are often not analytically known. Although a detailed discussion of the spot sizes of clipped beams is beyond the scope of this paper, we want to compare the beam’s spot size with the chosen lateral ranges. In the near field behind the aperture, i.e., with a Fresnel number F≫1, the spot size of the clipped beam is still roughly equal to the aperture radius, and so in our example (F=47.0), the spot size is approximately 0.5 mm. Therefore, in row one in Figure 1, the lateral range we show is 1.5 mm, which is approximately three times the spot size. For a propagation distance of 1000 mm, which is in the far field, we can use Equation (8) in [48] to estimate that the spot size of the clipped beam is 0.875 mm. This is consistent with the spot size of a Gaussian beam with a 0.5 mm waist at a propagation distance of 1000 mm, which is 0.842 mm, and, therefore, slightly smaller than the clipped beam, as expected. Yet, in our computation, 3 times 0.875 mm by no means met the requirement of propagation distance-independent DNMSE, so we had to extend the lateral range to 180 mm instead.

These large lateral distances, with a constant MEM beam power, result in the expected propagation distance Independence of the discretized normalized mean-square error $\varepsilon^{\mathrm{DNMSE}}$, as shown in the fourth column of Table 2, with only minor variations observed. We can see from Figure 1 that for propagation distances of 20 mm and beyond, there is a maximum lateral range wherein the phase is correctly approximated by the MEM (indicated by the zero lines for larger lateral distances). This is due to the finite size of the modes used in the decomposition. For a propagation distance *z*, the spot size of the higher-order modes along *x* and *y* is [49]

(22) $$\begin{array}{c} w_{x,mn}\left(z\right)=\sqrt{2m+1}w\left(z\right)=\sqrt{2m+1}w_{0d}\sqrt{1+{\left(z/z_{r}\right)}^{2}} \end{array}$$

(23) $$\begin{array}{c} w_{y,mn}\left(z\right)=\sqrt{2n+1}w\left(z\right)=\sqrt{2n+1}w_{0d}\sqrt{1+{\left(z/z_{r}\right)}^{2}} \end{array}$$

with w(z) being the spot size of the fundamental mode HG${}_{00}$ used in the decomposition and the Rayleigh $z_{r}=\pi w_{0d}^{2}/\lambda$. Using these equations, we can estimate the spot size of the highest mode used in the MEM. The MEM can, in turn, only resolve fields within a range of up to three times the spot size of this highest mode. We can show this with the example of a propagation distance of 1000 mm (lowest row in Figure 1) and a mode order of 50. For this, we find $w_{x,50\;0}=w_{y,0\;50}=32\mathrm{mm}$, resulting in a maximal resolvable range of approximately 96 mm, which precisely fits the observation in the phase graph.

It may be expected that a higher mode order automatically implies that a larger lateral range can be resolved. However, this is not necessarily the case, as can be seen in Figure 1 for a propagation distance of 20 mm. Here, the situation is reversed: the higher the mode order, the smaller the resolvable lateral range. This is a consequence of using Equation (19) to compute the optimal waist size, which decreases with increasing mode order.

Outside the maximal resolvable lateral range, the MEM fails and generates zero amplitudes and phases. Consequently, the relative error is approximately 1 outside the maximal resolvable lateral range. This is clearly visible in Figure 1. However, the large lateral range is not a choice usually taken in simulations since the spot properties are barely visible in these lateral ranges. Instead, simulations are usually performed with smaller lateral ranges in the target plane, as shown in Figure 2. Here, the lateral ranges cover only 0.4, 0.2, 0.06, and 0.022 times the ranges shown in Figure 1. For instance, for a propagation distance of 5 mm, the lateral distance shown in Figure 2 is 0.6 mm compared to the calculated spot size of 0.5 mm. For a propagation distance of 1000 mm, the computed spot size is 0.875 mm, in comparison to the 4 mm lateral distance shown in Figure 2. These lateral changes were chosen simply for good visualization of the amplitude and phase profiles without any hard criterion.

In the first row in Figure 2, one can see that the MEM with the given settings and mode orders of up to N=50 insufficiently resolves the high-frequency spatial oscillation in the very near field behind the aperture. However, the further the beam propagates, the better the performance of the MEM, such that after 1000 mm (i.e., at F=0.235), the wavefront is well represented, even with a mode order of 10. So, although the NMSE is propagation distance-independent and, therefore, constant for any choice of *N*, we can see in the left and center columns in Figure 2 how the precision of the MEM increases with increasing propagation distances. This means that the error radially transmits outwards and, therefore, may be in a radial distance of no interest to the application. This also shows that it is not always necessary to choose high mode orders, particularly in far-field simulations. Instead, the mode order should be chosen as a compromise between different criteria. The primary criterion is the increasing computational effort with increasing mode order. A second criterion is that the evaluation of the sum of Hermite–Gaussian modes with high polynomial orders is a typical mathematical challenge, resulting in numerical errors for high mode orders. Finally, the optimal decomposed beam waist calculated according to [22] and Equation (19) decreases with increasing mode order and needs to be sufficiently large to not violate the paraxial approximation. Consequently, the mode order should be chosen carefully under consideration of the intended precision and the costs and risks if the mode order chosen is too high.

We can now compare the different errors for the case of large lateral ranges. In these cases, the DNMSE (fourth column of Table 2) is propagation distance-independent and deviates from the analytically computed NMSE (third column) only slightly, with a maximum deviation of 9.32% (at 1000 mm with mode order 50). It can be seen that both the NMSE and DNMSE decrease with increasing mode orders for all propagation distances, as expected. In contrast to the DNMSE, the summed relative error $\varepsilon_{\sum }^{\mathrm{rel}}$ shown in column 6 is not propagation distance-independent but increases for any mode order with the propagation distance. However, for any propagation distance, the summed relative error decreases with increasing mode orders. For smaller lateral ranges, the DNMSE (fifth column of Table 2) is propagation distance-dependent, as indicated previously, but decreases with increasing mode orders for any given propagation distance, similar to the NMSE. Generally, it also decreases with increasing propagation distances for a given mode order but not strictly monotonously. Similarly, the summed relative error shown in column 7 is also propagation distance-dependent and decreases with increasing mode orders for any propagation distance. For any given mode order, it increases as the beam propagates due to the increasing step sizes Δr.

Concerning the various error definitions, we find that the DNMSE is not propagation distance-independent in typical simulation scenarios because the lateral range chosen is too small. The relative error we have introduced here is a useful quantity that allows for the qualitative evaluation of the performance of the MEM directly from a graph. For instance, it allows us to directly see in Figure 2) that the accuracy of the MEM increases with increasing mode orders. This aligns with the findings from the DNMSE and NMSE, but only in numbers that cannot be visualized comparably. In cases where the relative errors cannot be clearly distinguished from the graph, e.g., in the first row in Figure 2, the summed relative error can help quantify the physical dependencies (like the performance change with the mode order or propagation distance). However, one should always keep in mind the non-ideal properties of the summed relative error, especially its dependence on the selected lateral range (see Figure 1 and Figure 2).

In conclusion, we find that all the defined types of errors have their strengths and weaknesses, such that a comparison of the performance of the MEM with the different error types can be helpful. Concerning the MEM itself, we find that it does not ideally resolve the high spatial oscillations of the diffracted beam in the near field but accurately describes the beam in the far field, even when only low mode orders are used.

### 2.2. Properties and Individual Test of the Gaussian Beam Decomposition

#### 2.2.1. GBD: Method Description

**Figure 3:**
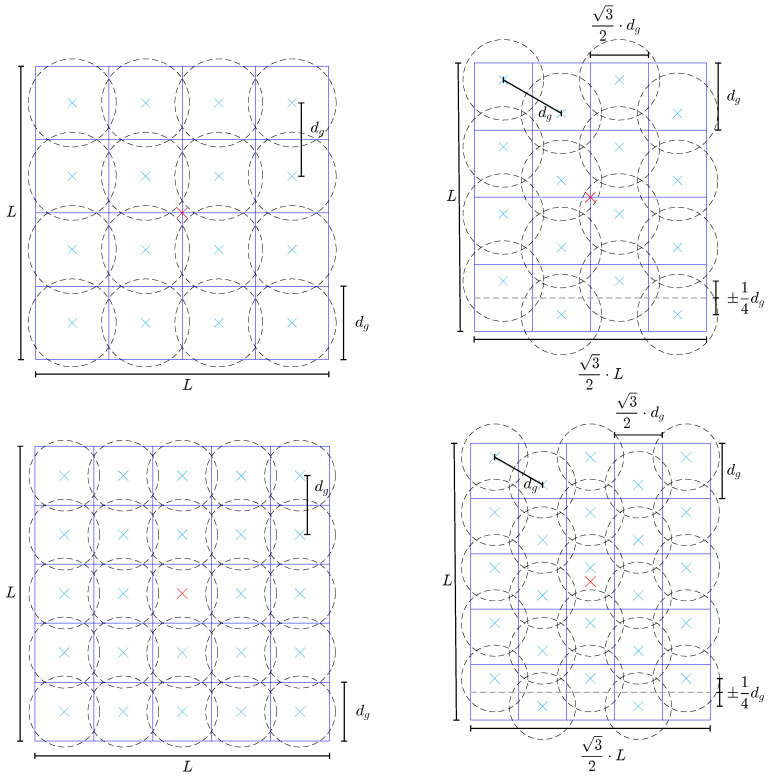
Illustration of the GBD method. On the left is the square grid structure, and on the right is the hexagonal grid structure, for even and odd *g*, respectively. One can see the (virtual) grid in blue, with the center marked in red. The origin points of the individual grid beams are marked in cyan, and their waists with radius $w_{0g}$ are shown as dashed circles. The waist scaling factor $f_{\mathrm{ws}}$ was set to 1.2 in the diagrams.

**Figure 4:**
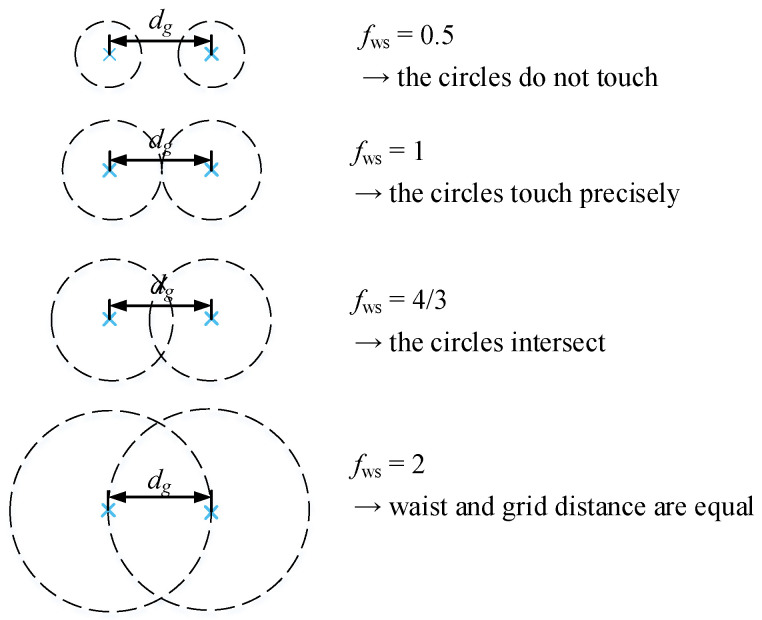
Illustration of the waist scaling factor $f_{\mathrm{ws}}$. If $f_{\mathrm{ws}}=0.5$, the grid beams do not touch. If $f_{\mathrm{ws}}=1$, the grid beams precisely touch. If $f_{\mathrm{ws}}=\frac{4}{3}$, the grid beams intersect. If $f_{\mathrm{ws}}=2$, the grid beams’ waist radii and grid distances are equal.

Similar to the MEM, the GBD is a wavefront decomposition method. However, it decomposes any wavefront into fundamental Gaussian beams on a grid, as illustrated in Figure 3. There are currently two supported shapes for the decomposition grid: square or hexagonal. Both grid shapes are depicted in Figure 3. The quantities that define the grid are the edge length *L*, known as the window size, and the number of fundamental Gaussian beams along each dimension *g*. The lattice constant $d_{g}$, known as the grid distance, is defined as $d_{g}=\frac{L}{g}$. The images show the grid of fundamental Gaussian beams, depicted here by dashed circles that denote their waist radius $w_{0g}$. The waist radius is defined as

(24) $$w_{0g}=f_{\mathrm{ws}}\;\cdot \;\frac{d_{g}}{2}=f_{\mathrm{ws}}\;\cdot \;\frac{L}{2g}\;,$$

where $f_{\mathrm{ws}}$ is the so-called waist scaling factor. For $f_{\mathrm{ws}}=1$, the waists exactly touch each other. For larger $f_{\mathrm{ws}}$, the overlap of the fundamental Gaussian beams increases; for smaller $f_{\mathrm{ws}}$, it decreases, as shown in Figure 4. The number of grid beams along each dimension is denoted as *g*, with g×g=G being the total number of grid beams placed within the window, as shown in Figure 3. We refer to this total number of grid beams as the grid size. The definitions of the window size and waist scaling factor shown here are the same as those in the IfoCAD; however, other software could use different definitions.

The hexagonal grid can be directly constructed from the square grid without changing the number of points or underlying math. Therefore, the algorithm used to compute the GBD can remain unchanged when switching between grid geometries. To construct the hexagonal grid, columns with an even index are shifted up by $\frac{d_{g}}{4}$ relative to the square grid position, and columns with an odd index are shifted down by the same amount to create a hexagonal point structure. A rescaling by a factor of $\frac{\sqrt{3}}{2}$ along the horizontal direction is required to create an equidistant separation between the nearest neighbors of points, thus forming equilateral triangles. The mapping can be described by the function

(25) $$\begin{array}{cc} x_{ij}^{\prime } & =\frac{\sqrt{3}}{2}\left(x_{ij}-x_{0}\right)+x_{0}\\ y_{ij}^{\prime } & =\left\{\begin{array}{c} y_{ij}+\frac{d_{g}}{4}\;\mathrm{if}\;i\;\mathrm{is}\;\mathrm{even},\\ y_{ij}-\frac{d_{g}}{4}\;\mathrm{if}\;i\;\mathrm{is}\;\mathrm{odd} \end{array}\right. \end{array}$$

where $x_{ij}^{\prime }$ and $y_{ij}^{\prime }$ are the coordinates of the ij-th grid point, calculated from the coordinates of the square grid point $x_{ij}$ and $y_{ij}$, and $x_{0}$ is the x-coordinate of the grid center. However, this transformation shrinks the window size in the *x*-direction due to the rescaling, resulting in a new window size of $\frac{\sqrt{3}}{2}L\times L$. The waist size $w_{0g}$ is the same as for the square grid because the nearest neighbor distance remains $d_{g}$ in both cases.

In this paper, we mostly use the square grid and only use the hexagonal grid in one particular case in Section 2.2.4. Therefore, the mathematical description below focuses on the square grid because the basic theory remains the same for both grids. The goal of the GBD is to represent the wavefront as a superposition of the fundamental Gaussian grid beams weighted by coefficients:

(26) $$E\left(x,y\right)\approx \sum_{i=0}^{g}\sum_{j=0}^{g}b_{ij}E_{ij}\left(x-x_{ij,0},y-y_{ij,0}\right),$$

where *E* is the continuous wavefront to be decomposed, $E_{ij}$ are the electric fields of the grid beams with unity intensity, $\left(x_{ij,0},y_{ij,0}\right)$ are their origin points, and $b_{ij}$ are the complex weighting coefficients. To determine the coefficients, the above equation is evaluated at a discrete set of sampling points $\left(x_{k},y_{l}\right)$, where *k* and *l* describe the location on the grid: one is the column, and the other is the row. The resulting linear equation system is then solved for $b_{ij}$. There must be at least as many sampling points as there are coefficients, which is *G*, one sampling point per grid beam. For better precision, one can also choose more sampling points. But because only the minimal number of required sampling points was used in the simulations in this paper, we focus our explanations on this case.

Both pairs of indices are compressed into a single sequential index to be able to write the linear equation system in matrix form. $E(x_{k},y_{l})$ and the $b_{ij}$ can be written as column vectors, $\vec{W_{s}}$ and $\vec{b}$, respectively. $E_{ij}\left(x_{k}-x_{ij,0},y_{l}-y_{ij,0}\right):=m_{ijkl}$ can be interpreted as a matrix *M*, with each row corresponding to a sampling point containing the electric fields of each grid beam at this sampling point. These matrix entries describe how strongly each grid beam influences the value of the superimposed electric field in the sampling point. Therefore, the GBD can be expressed as

(27) $$\vec{W_{s}}=M\vec{b},\;$$

which can be solved using well-understood methods such as QR decomposition, which is adopted in IfoCAD. The following equations show in detail how $\vec{W_{s}}$, $\vec{b}$, and *M* are composed for an equal number of grid beams and sampling points of *G*.

(28) $$\begin{array}{c} \vec{W_{s}}=\left(\begin{array}{c} E(x_{1},y_{1})\\ \vdots \\ E(x_{1},y_{g})\\ \vdots \\ E(x_{k},y_{l})\\ \vdots \\ E(x_{g},y_{1})\\ \vdots \\ E(x_{g},y_{g}) \end{array}\right),\;\vec{b}=\left(\begin{array}{c} b_{11}\\ \vdots \\ b_{1g}\\ \vdots \\ b_{ij}\\ \vdots \\ b_{g1}\\ \vdots \\ b_{gg} \end{array}\right)\;. \end{array}$$

(29) $$M=\left[\begin{array}{ccccc} m_{1111} & \cdots & m_{ij11} & \cdots & m_{gg11}\\ \vdots & \cdots & \vdots & \cdots & \vdots \\ m_{11kl} & \cdots & m_{ijkl} & \cdots & m_{ggkl}\\ \vdots & \cdots & \vdots & \cdots & \vdots \\ m_{11gg} & \cdots & m_{ijgg} & \cdots & m_{gggg} \end{array}\right]$$

The GBD can be computationally expensive if large grid sizes are chosen. For example, if G=1000×1000, $10^{6}$ beams will be superimposed, so both ${\vec{W}}_{s}$ and $\vec{b}$ have $10^{6}$ entries, which makes *M* of size $10^{6}\times 10^{6}$. Another approximation is employed to further reduce the complexity of the problem. Gaussian beam intensities drop off rapidly with increasing distances from the center. At points a few waist sizes apart, their contribution is near zero. Therefore, if the distance between the sampling point and the grid beam origin is larger than $3w_{0g}$, the beam’s contribution to the electric field at the sampling point is negligible. The corresponding element in *M* can be set to zero. Consequently, *M* becomes a sparse matrix, and a software implementation making use of this can reduce both memory consumption and computational effort. Nonetheless, the high dimensionality of the equations should be kept in mind when choosing the grid size of a GBD. The mathematical form of the sparsification can be expressed by

(30) $$m_{ijkl}=\left\{\begin{array}{cc} m_{ijkl} & \mathrm{if}\;d_{ijkl}\leq 3w_{0g}\\ 0 & \mathrm{else}\;, \end{array}\right.$$

where $d_{ijkl}$ is the distance between the grid beam origin $\left(x_{ij,0},y_{ij,0}\right)$ and the sampling point $\left(x_{k},y_{l}\right)$.

#### 2.2.2. GBDs and Their Errors

To assess the quality and performance of the GBD, we define a comparable set of errors, similar to the MEM. An NMSE for the GBD, the same as in Equation (11), can be defined but not evaluated analytically as in the MEM (Equation (12)):

(31) $$\begin{array}{c} \varepsilon^{\mathrm{NMSE},\mathrm{GBD}}:=\frac{{∥E^{\mathrm{GBD}}-E∥}^{2}}{{∥E∥}^{2}}=\frac{{∥E^{\mathrm{GBD}}-\left(E^{\mathrm{GBD}}+E^{\mathrm{err}}\right)∥}^{2}}{{∥E∥}^{2}}=\frac{P^{\mathrm{err}}}{P}\;. \end{array}$$

Here, we assume that the exact field E=E(x,y,z) can be split into the GBD representation $E^{\mathrm{GBD}}=E^{\mathrm{GBD}}\left(x,y,z,w_{0g},L,f_{ws}\right)$ and a residual field $E^{\mathrm{err}}=E^{\mathrm{err}}\left(x,y,z\right)$, and we assume that this residual field comprises a power of $P^{\mathrm{err}}$. Unlike in the MEM case, we cannot evaluate $P^{\mathrm{err}}$, particularly because $E^{\mathrm{err}}$ is not orthogonal to $E^{\mathrm{GBD}}$, which means that these fields interfere. However, we can assume that $P^{\mathrm{err}}$ is independent of the propagation distance, provided that propagation through a vacuum is assumed, where no losses and no energy exchange with a medium occur. Likewise, the power of the entire field is conserved during propagation, such that we see in Equation (31) that the NMSE of the GBD is as propagation distance-independent as the NMSE of the MEM.

Additionally, we can define the discretized NMSE similarly to the MEM error defined in Equations (13) and (14), as follows

(32) $$\varepsilon_{\circ }^{\mathrm{DNMSE}}\left(N_{R},R,z\right):=\frac{\sum_{i=0}^{N_{R}}2\pi {\left|M\vec{b}\left(r_{i},z,L,w_{0g},f_{\mathrm{ws}}\right)-\vec{W_{s}}\right|}^{2}r_{i}\Delta r}{P}\;.$$

(33) $$\varepsilon_{□}^{\mathrm{DNMSE}}\left(N_{X},N_{Y},X,Y,z\right):=\frac{\sum_{i=1}^{N_{X}}\sum_{j=1}^{N_{Y}}{\left|M\vec{b}\left(x_{i},y_{j},z,L,w_{0g},f_{\mathrm{ws}}\right)-\vec{W_{s}}\right|}^{2}\Delta x\Delta y}{P}\;.$$

Similarly, we define the 2D and 1D versions of the relative error, as well as the summed relative error for the GBD:

(34) $$\varepsilon^{\mathrm{rel}}\left(x_{i},y_{i},z\right):=\frac{\left|M\vec{b}\left(x_{i},y_{i},z,L,w_{0g},f_{\mathrm{ws}}\right)-\vec{W_{s}}\right|}{\left|\vec{W_{s}}\right|}\;,$$

(35) $$\varepsilon^{\mathrm{rel}}\left(r_{i},z\right)=\frac{\left|M\vec{b}\left(x_{i},0,z,L,w_{0g},f_{\mathrm{ws}}\right)-\vec{W_{s}}\right|}{\left|\vec{W_{s}}\right|}\;,$$

(36) $$\begin{array}{cc} \varepsilon_{\sum }^{\mathrm{rel}}\left(N_{X},N_{Y},X,Y,z\right): & =\sum_{i=1}^{N_{X}}\sum_{j=1}^{N_{Y}}\frac{\left|M\vec{b}\left(x_{i},y_{j},z,L,w_{0g},f_{\mathrm{ws}}\right)-\vec{W_{s}}\right|}{\left|\vec{W_{s}}\right|}\Delta x\Delta y\\ \; & =\sum_{i=1}^{N_{X}}\sum_{j=1}^{N_{Y}}\varepsilon^{\mathrm{rel}}\left(x_{i},y_{j},z\right)\Delta x\Delta y\;, \end{array}$$

(37) $$\varepsilon_{\sum }^{\mathrm{rel}}\left(N_{R},R,z\right):=\sum_{i=1}^{N_{R}}\frac{2\pi \left|M\vec{b}\left(r_{i},z,L,w_{0g},f_{\mathrm{ws}}\right)-\vec{W_{s}}\right|r_{i}\Delta r}{\left|\vec{W_{s}}\right|}\;,$$

We have now defined the same type of errors for the GBD as for the MEM, and we can use these for comparison. However, we do not know any major characteristics of the given errors when applied to a GBD. Therefore, we study and discuss their characteristics in the examples provided throughout this paper.

#### 2.2.3. GBD Settings

For the MEM, it is known that for the stated set of applications, the relation defined in Equation (19) can be used to achieve minimal error in the decomposition. For the GBD, we could not find any comparable information. Since it is unclear how the parameters of the GBD should be chosen for minimal error, we can only state fairly general information and the typical settings we chose in our simulations.

For the current implementation of the GBD in IfoCAD, the parameters that can be chosen explicitly are the waist scaling factor $f_{\mathrm{ws}}$, the number *g* of grid beams along each primary axis of the square grid, and the window size *L*. The waist radius $w_{0g}$ of the grid beams and grid distance $d_{g}$ are then determined using Equation (24). Therefore, there are three parameters that influence the precision of the decomposition, among which the one-dimensional number *g* of grid beams roughly compares with the mode order *N* of the MEM. One intuitively expects that for a fixed window size, the larger the number of grid beams *g*, the higher the precision, although this is valid only within a certain range. We discuss this property in Section 2.2.4. Furthermore, in Section 3, we investigate how to choose the grid length *g* and mode order *N* if both methods are being directly compared. Therefore, we understand the number *g* of grid beams as the primary handle for the precision of the GBD. The remaining two parameters (the waist scaling factor $f_{\mathrm{ws}}$ and the window size *L*) are secondary handles, and we discuss their settings below.

In this paper, we use two different types of examples: a non-clipped Gaussian beam being decomposed or a wavefront clipped by an aperture. In the first example, the window size needs to be at least three times larger than the waist size; otherwise, the beam would be clipped by the window during the decomposition, resulting in unintended and unphysical diffraction. On the other hand, the window should not be chosen too large to avoid an unnecessarily high number of grid beams with zero amplitudes and no influence on the final result. Comparable arguments hold for the second type of example. Here, the window size needs to be sufficiently larger than the aperture. If the window is chosen to be smaller than the aperture size, the beam would obviously be clipped by the window rather than the aperture. If the window size is chosen to be equal to the aperture size or only slightly larger than it, the GBD would not be able to resolve the step function in the electric field, resulting in a GBD beam with a considerable residual electric field amplitude outside the window. Only if the window is sufficiently oversized (compared to the aperture) can the grid beams resolve the step function in the electric field amplitude that originates from the clipping aperture and thereby accurately decompose the entire wavefront of interest. For the examples in this paper, the window size is chosen to be between 1 to 1.5 times the diameter of the aperture. A window size equal to the diameter of the aperture is only used in the examples of non-clipped Gaussian beams (see Section 4.1), as in these cases, the diameter of the aperture is already sufficiently large and does not clip the beam. Please note that the window size is defined as a full width, rather than comparing it to the diameter of the aperture.

The waist scaling factor should be chosen such that the grid beams have a non-negligible overlap. If the waist scaling factor is chosen to be too small, the GBD could accurately resolve the incident electric field in the grid points, but due to the lack of overlap of the grid beams, the GBD beam would effectively have ‘holes’ between the grid points. On the other hand, if the waist scaling factor is chosen to be very large, a high number of grid beams would contribute to the electric field in every sampling point, thereby significantly increasing the computational effort. In this paper, for all examples, the range of $f_{\mathrm{ws}}$ is chosen to be between 3/2 and 10/3.

Unfortunately, we do not know of an analytic relation between grid size, window size, and waist scaling factor that forms an ideal choice for typical decompositions. The waist scaling factors and window sizes chosen in this paper are not strictly optimized for the given examples but simply follow the given logic.

#### 2.2.4. Example: GBD Performance for a Clipped Gaussian Beam

In this subsection, we illustrate two examples of the performance of the GBD. In both cases, we decompose a Gaussian, which is clipped by a circular aperture. We assume normal incidence and the Gaussian beam to be optimally centered on the aperture. In the first example, we investigate the performance of the GBD with increasing grid size and compare and test the different error definitions. The second example illustrates the behavior of square and hexagonal grid shapes for the same grid size. Like the MEM example, the two examples here meet the assumption of the paraxial approximation.

##### Example 1: Comparing Different Grid Sizes

For the MEM, it is known from analytic equations that the precision of the decomposition increases monotonously with increasing mode order *N*. Consequently, this is also observed in the simulations, as long as the numerical errors are sufficiently small. For the GBD, one may likewise want to assume that for a fixed window size, the precision of the GBD increases with an increasing number of grid beams. However, as we showed in Section 2.2.1 and Equation (24), the waist $w_{0g}$ of the grid beams scales inversely with the number *g* of grid beams. This means that the more grid beams are used, the smaller the grid beams’ waists will become, provided that the waist scaling factor is not adapted. Therefore, an increasing number of grid beams can quickly result in a violation of the paraxial approximation. Therefore, it cannot be generally expected that an increasing number of grid beams will increase the precision of the decomposition.

In this example, we intentionally work with a fixed window size *L* and a fixed waist scaling factor $f_{\mathrm{ws}}$ and increase the grid size *G* up to values that cause the waist sizes to be in the order of the wavelength, thereby violating the paraxial approximation assumption. With this, in one simple example, we test how the precision changes with the grid size, and we test slightly beyond settings that would normally be chosen.

The parameter settings of this example are listed in Table 3. In this example, the beam parameter, aperture size, shape and alignment, propagation distances, and sampling points are all chosen to be the same as in the MEM example in Section 2.1.4. The grid sizes are 100×100, 200×200, 500×500, and 1000×1000, respectively, using a square grid with a window size of 1.5 mm and a waist scaling factor of $f_{\mathrm{ws}}=1.5$. The grid beam waist $w_{0g}$ is calculated using Equation (24). As shown in Table 3, the resulting waist sizes are critically small and up to a clear violation of the paraxial approximation in the case of 1000×1000 grid beams.

**Figure 5:**
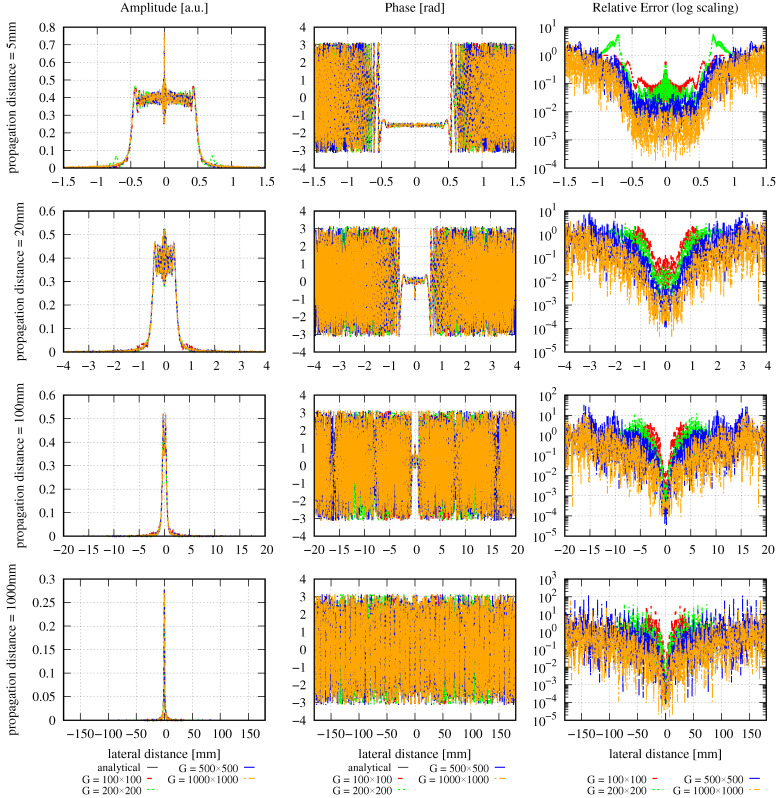
Performance of the GBD for large lateral ranges with different grid sizes (G = 100×100, 200×200, 500×500, and 1000×1000) for an incoming circular Gaussian beam with a 2 mm waist being clipped by a 0.5 mm radius circular aperture. The amplitude (absolute value), phase, and relative errors $\varepsilon^{\mathrm{rel}}\left(N_{R},R,z\right)$ are shown at different propagation distances of z= 5 mm, 20 mm, 100 mm (near field) and 1000 mm (far field) after the clipping aperture. The analytical methods used for the near and far fields were those of Campbell [46] and Tanaka et al. [47], respectively. The lateral distances for each propagation distance were chosen to be large enough to cover all the power. For any propagation distance, the performance of the GBD improves with the increasing grid size.

**Figure 6:**
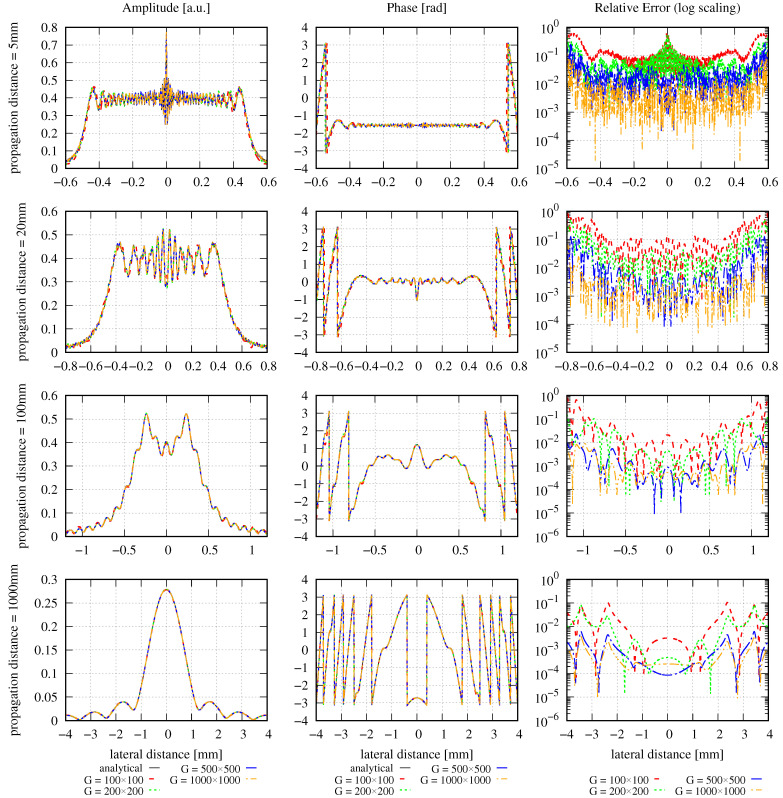
Same as Figure 5 but for smaller lateral ranges. The performance of the GBD with different grid sizes is shown for an incoming circular Gaussian beam with a 2 mm waist being clipped by a 0.5 mm radius circular aperture. The amplitude (absolute value), phase, and relative errors $\varepsilon^{\mathrm{rel}}\left(N_{R},R,z\right)$ are shown at different propagation distances of z= 5 mm, 20 mm, 100 mm (near field) and 1000 mm (far field) after the clipping aperture. The analytical methods used for the near and far fields were those of Campbell [46] and Tanaka et al. [47], respectively. One can see that the further the beam propagates or the larger the grid size, the better the performance of the GBD.

The amplitude, phase, and relative errors are plotted for large and small lateral ranges in Figure 5 and Figure 6, respectively. The corresponding errors are summarized in Table 4. Since the electric field of interest is the same as in the MEM example, we use the same lateral ranges as in Section 2.1.4.

Like in the MEM example shown in Figure 1, the GBD results with the large lateral ranges shown in Figure 5 are not very descriptive because the lateral ranges are simply too large to assess the quality of the decomposition. However, Figure 6 shows that the GBD effectively describes the beam, particularly in the far field, even if only 100×100 grid beams are used. Additionally, it can be seen that the further the beam propagates, or the larger the grid size, the better the performance of the GBD. This is also reflected in both the DNMSE (in column 4) and the summed relative error (in column 6), as they decrease with increasing grid sizes, although not strictly monotonously for increasing propagation distances with a given grid size in column 6. This is particularly interesting, given that the large grid sizes imply that unadvisably small waist sizes were used. The observed increasing precision of the GBD with these large grid sizes is, therefore, not naturally given. Additionally, the summed relative error in column 6 also changes nonmonotonically as the diffracted beam propagates.

As mentioned above, the NMSE of the GBD is propagation distance-independent when the power of the entire field is conserved during propagation (see Equation (31)). However, in column 3 in Table 4, it can be seen that the DNMSE of the GBD is propagation distance-dependent in the case of large lateral ranges, and the reason for this behavior comes from the non-ideal choice of the lateral ranges. Additionally, it can be seen that the DNMSE decreases with increasing grid sizes for any given propagation distance. This holds for both choices of lateral ranges. However, it is not consistently given that for any choice of grid size, there is a strictly monotonous decrease in the DNMSE with increasing propagation distances. This again differs from the behavior of the MEM. However, it is currently unclear whether this originates from the method itself or its implementation. Likewise, the summed relative error $\varepsilon_{\sum }^{\mathrm{rel}}$ does not show a strictly monotonous decrease with increasing grid sizes. For example, the summed relative error in column 5 at a propagation distance of 5 mm increases in the step from a grid size of 100×100 to 200×200, and decreases for any further increase in the grid size. Finally, the summed relative error in column 5 increases with the propagation distance, unlike the DNMSE, which mostly decreases with the propagation distance. The reason for these observations could again be due to the method itself, numerical precision, implementation problems, or the nearly zero-valued denominator in the error computation. Despite the nonmonotonous behavior of the DNMSE and the summed relative error, we still used both for the total performance evaluation of the GBD to facilitate a direct comparison with the MEM.

##### Example 2: Comparing Grid Shapes

**Figure 7:**
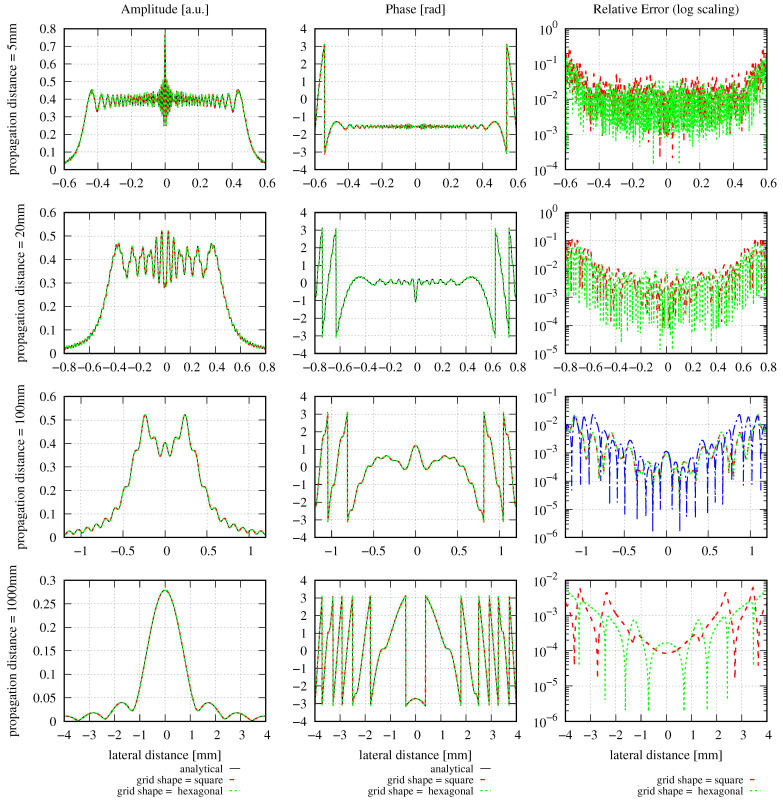
Performance of the GBD with hexagonal and square grid shapes. The amplitude (absolute value), phase, and relative error distributions are shown at different propagation distances from a circular aperture with a radius of 0.5 mm. The incoming circular-symmetric Gaussian beam was centered to the aperture and had its 2 mm waist located in the aperture plane. The analytical methods used for the near and far fields were those of Campbell [46] and Tanaka et al. [47], respectively. The number of sampling points *X* is 3001.

The shape of the grid can affect the accuracy of the GBD. Therefore, we repeat the previous example with the same settings and a grid size of 500×500 beams but this time, we compare the performance of the GBD to a square and a hexagonal grid. As introduced in Section 2.2.1, the window size in the horizontal direction is rescaled by a factor of $\sqrt{3}/2$ for the hexagonal grid, which is 1.5 mm $\times \sqrt{3}/2$ in this example. We use the same waist scaling factor $f_{\mathrm{ws}}=1.5$ and waist radius $w_{0g}$ for both the square and hexagonal grids in IfoCAD. The resulting amplitude, phase, and relative errors of the square and hexagonal grids are plotted in Figure 7. The corresponding DNMSE and summed relative error are listed in Table 5.

Both Figure 7 and Table 5 show that at propagation distances of 5 mm and 20 mm, the simulations performed using the hexagonal grid gradually show slightly better results compared to the simulations performed using a square grid. However, at larger distances of 100 mm or 1000 mm, the square grid resulted in higher accuracy. However, this is only one example, and we cannot draw a generalized conclusion from it.

## 3. Fair Comparison

Thus far, the MEM and GBD have been introduced, their settings discussed, and their performance individually tested using an example. In the next step, we aim to directly compare the two methods. To do so, we need to establish criteria for evaluating their performance and determining if one method outperforms the other or if they perform equally well. Specifically, we need to define which mode order of the MEM should be compared to which grid size of the GBD, and provide the rationale for this choice. This is discussed below.

**Criteria for a fair comparison**

There are two aspects that should be considered when comparing the MEM and GBD: accuracy and computational effort. We assess accuracy based on the introduced errors and computational effort by the runtime of the simulation. We consider a fair comparison to be a case where either both methods achieve the same accuracy, and then the runtime is used to assess the performance, or, both methods are set to have approximately the same runtime, and the performance is assessed by the achieved accuracy. In this study, we use the latter criterion and choose the elapsed real time (not the CPU time) as the runtime.

From the basic principles of the two methods, the computational effort comprises three parts: decomposition time, propagation time, and superposition time. For the MEM, the decomposition time is the time spent on the integration to calculate the coefficients of the higher-order modes. For the GBD, it is the time needed for the QR decomposition to solve Equation (27). Therefore, the decomposition time depends not only on the mode order and grid size but also on the properties of the input field. The propagation time is very short in comparison because ray-tracing methods, including the propagation of the Gaussian beam parameters with the ABCD matrix formalism, are highly efficient and computationally undemanding. For the MEM, all modes even share the same axis, such that only one ray needs to be traced, which then represents the beam axis of all modes. Finally, the superposition time depends on the number of sampling points in the target plane, as well as the number of modes or grid beams. Therefore, the computational effort is dominated by the decomposition and superposition times but is also naturally affected by other criteria, such as the efficiency of the original implementation of the methods in the software tool used, the computational power of the computer used, and possibly even the operating system used.

In this study, we compare the performance of the MEM to that of the GBD using IfoCAD (version 2022/10, git commit adf19a5b) to find mode orders and grid sizes that result in similar computational efforts. All simulations shown here for testing the computational efforts were performed on a MacBook Pro 2020 with 8 GB of RAM and a 2.3 GHz processor with 8 cores.

**Computational effort of the MEM and GBD**

**Figure 8:**
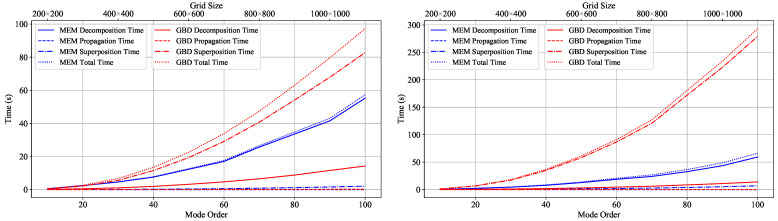
(**Left**) Computational efforts of the MEM and GBD describing the electric field of a clipped Gaussian beam propagated to a target plane, sampled along 3001 points along the *x*-axis (y=0), where the total CPU time of the GBD is around 1.23 times that of the MEM. (**Right**) Computational efforts of the MEM and GBD, using the same settings as in the graph on the left, except for the use of 101×101 sampling points in the xy-plane, where the total CPU time of the GBD is around 3.53 times that of the MEM. Both graphs indicate that the MEM is better parallelized compared to the GBD in IfoCAD.

In order to find settings that result in comparable computational efforts for both methods, we performed a dedicated simulation where we varied the mode order and grid size while keeping the number of sampling points in the target plane constant. For this simulation, we once again chose the case of a clipped Gaussian beam, using the same settings as in the previous examples: a circular Gaussian beam with a waist of 2 mm located at the aperture center, incident onto this circular aperture with a radius of 0.5 mm. We set *N* to range from 10 to 100 in increments of 10 for the MEM and set *g* to range from 100 to 1000 in increments of 100. The target plane was 5 mm away from the aperture, and we used 3001 sampling points to compute the electric field for x∈−3,3,y=0. In this case, both the MEM and GBD were run with parallelization. Therefore, we distinguish between two different times: the elapsed real time, which is the time the user needs to wait for a result, and the CPU time, which is the actual computation time and is longer than the elapsed real time due to the used parallelization. Here, we focus on the elapsed real time and use it as the primary criterion. The computational efforts of the MEM and GBD are summarized in the graph on the left in Figure 8, which contains information about the elapsed times (wall clock time) for decomposition, propagation, and superposition.

For the MEM, the decomposition time was by far the dominant time consumer in the given example, consuming more than 95% of the total elapsed real time. For the GBD, the situation was different: the superposition time was dominant over the decomposition time by a factor of more than 3. The propagation time, as expected, was insignificant. For GBDs with large grid sizes, some computational efforts accumulated due to the large number of grid beams that needed to be traced. For instance, for a grid size of 1000×1000, 1 million grid beams needed to be propagated. Finally, the different superposition times are noteworthy. We understand this was a consequence of the different numbers of beams that needed to be computed and evaluated at every target grid point. For instance, a mode order of 100 implies that 51×52/2=1326 modes were used in the MEM (cf. Equation (9)). In the case of the GBD, a grid size of 500×500 grid beams implies that in every sampling point of the target plane, 250,000 beams were being tested, whether or not they contributed to the electric field. Even though only a few electric fields were indeed being superimposed in the end, the test itself for the high number of grid beams cost considerable time in the current implementation.

If we compare the total elapsed real time for different settings of the MEM and GBD, we can see several pairings of grid size *G* and mode order *N* that can be used to achieve comparable computational efforts. For instance, {N=10, G=100×100}, {N=20, G=200×200}, and {N=50, G=400×400}. In this paper, we chose {N=50, G=400×400} for all comparisons using 3001 sampling points.

However, this choice depends on the number of sampling points used in the target plane, as the computational effort of the GBD is primarily influenced by the superposition time (which significantly depends on the number of sampling points in this plane), whereas this is not the case for the MEM. Therefore, the illustrated comparison should be repeated if a different number of sampling points is used. In this paper, we used 3001 sampling points for all two-dimensional cross-sections of the electric field. However, we also show figures for the full cross-sections (*x* and *y* for a fixed propagation distance *z*) and used 101×101 sampling points in these cases. Therefore, we repeated the above simulation for x∈−3,3,y=∈−3,3 with 101 points on each axis, i.e., 10201 sampling points in total. The results are shown in the graph on the right in Figure 8. Based on the computational analyses of the MEM and GBD conducted in this scenario, we selected N=50 and G=300×300 for simulations with 10201 sampling points, particularly when *y* was not set to 0 in this paper.

Indeed, these parameters resulted only in roughly comparable computational efforts, and better matching could be achieved if intermediate values were used. However, this was not necessary and was not the aim here since the computational effort depends on additional simulation parameters and will vary for other setups, particularly with regard to the properties of the wavefront that is to be decomposed, as well as the sampling grid in the target plane. However, our experience has shown that the configurations used consistently yielded comparable computational efforts for all simulations discussed in this paper.

Finally, we can compare the elapsed real times and CPU times, thereby achieving the parallelization of both methods. For comparable parameters, i.e., {N=50,G=400×400} and {N=50,G=300×300}, the CPU time of GBD was longer. This shows that the GBD was more strongly parallelized compared to the MEM in the IfoCAD version used.

## 4. Method Comparison

In this section, we directly compare the performance of the MEM and GBD for various scenarios, which include non-clipped and clipped Gaussian beams in free space (Section 4.1), aberrated wavefronts (Section 4.2), and reflection from optical components (Section 4.3).

### 4.1. Non-Clipped Gaussian Beams and Clipped Gaussian Beams in Free Space

In all comparisons in this subsection, we compute the introduced errors to evaluate the quality of each method. Unfortunately, this requires the electric field *E* to be known in every target plane, which strongly restricts the number of possible test cases. Therefore, in Section 4.1.1, we test the performance of the MEM and GBD for non-clipped circular and general astigmatic Gaussian beams, for which the analytic representation of the electric field is widely known. In Section 4.1.2, we further investigate the case of circular symmetric clipped Gaussian beams in the near, far, and extremely far fields, for which again, we can use the analytic representations provided by Campbell [46] and Tanaka et al. [47] in the Fresnel and Fraunhofer regions. For the extremely far field, we refer to propagation distances of a few million kilometers, which occur in space-gravitational wave detectors such as LISA [6] and Taiji [7].

#### 4.1.1. Non-Clipped Gaussian Beams

##### Circular Gaussian Beam

The simplest case used to compare the MEM and GBD is that of non-clipped Gaussian beams, for which the electric field is analytically known in any propagation distance. Therefore, we first perform a comparison of the MEM and GBD for the example of a non-clipped circular-symmetric Gaussian beam with the parameters listed in Table 6. It should be noted that we defined an aperture here. This is due to the IfoCAD version used, which requires an aperture to be defined for both methods. However, with the radius being four times larger than the Gaussian waist radius, it was effectively not clipping the beam, given that the clipped power was approximately $1.3\times 10^{-12}$ % of the full beam power. Instead, the aperture radius effectively defined the lateral range used in the numerical evaluation of the integral in Equation (7). In the GBD, we then used the same aperture radius to decompose the same input field as in the MEM case. We chose not to overscale the window further, and we set the window size (which is a full width) to be equal to the aperture diameter so as not to place an unnecessary number of grid beams in regions without field amplitudes.

**Figure 9:**
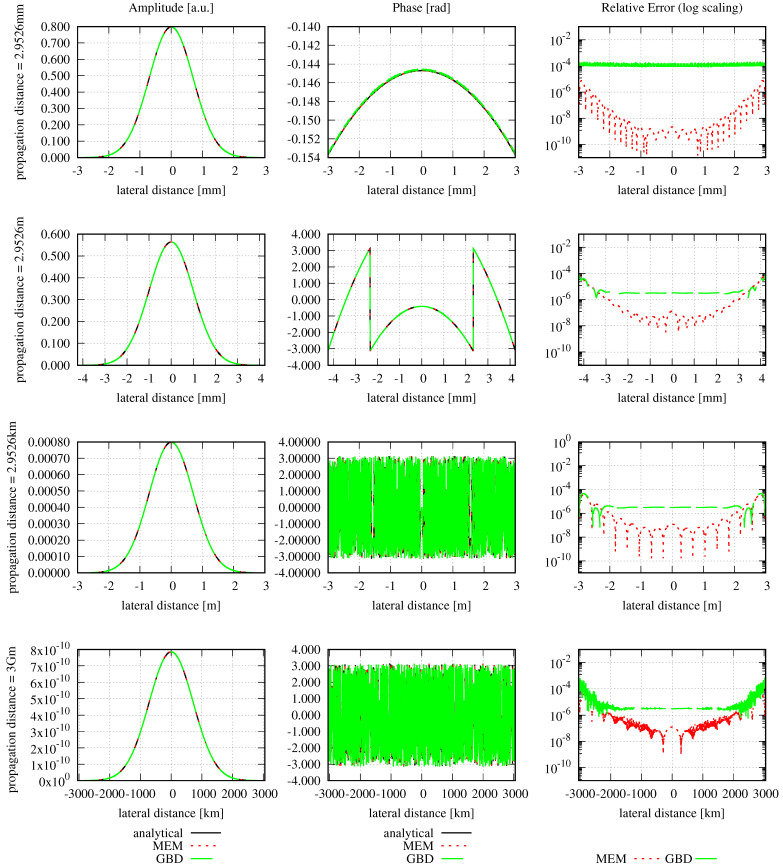
The amplitude (absolute value), phase, and relative error distributions at propagation distances of $z_{r}/1000$, $z_{r}$ (near field), $1000\;z_{r}$ (far field), and 3 million kilometers (extremely far field). The simulation parameters are listed in Table 6. The analytical results refer to the complex electric field of the non-clipped Gaussian beam in this case. It can be seen that the MEM behaved better than GBD.

**Figure 10:**
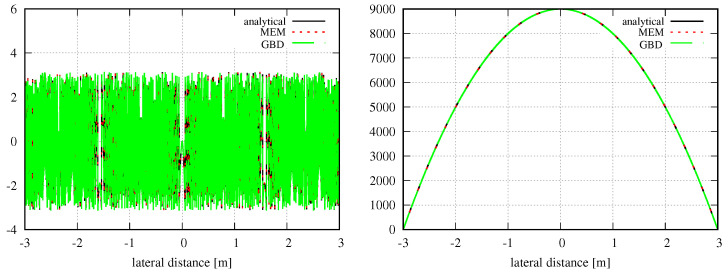
The wrapped and unwrapped phases of a propagation distance od 2.9526 km for the non-clipped circular Gaussian beam.

Concerning the optical setup, we defined a circular Gaussian beam with a waist radius of 1 mm, which was centered in this aperture. The mode order, grid size, and sampling points were chosen, as described in Section 3. The waist $w_{0d}$ of the modes used in the MEM was calculated using Equation (19); the GBD grid beam waist $w_{0g}$ was correspondingly calculated using Equation (24). After the decomposition, the MEM beam and GBD beam were propagated for $z_{r}/1000$, $z_{r}$, $1000\;z_{r}$, and 3 million kilometers (3 Gm), with $z_{r}=2.9526m$, where $z_{r}$ refers to the Rayleigh range of the incident Gaussian beam. We refer to the far field when $z\gg z_{r}$ [4]. The resulting amplitude, phase, and relative errors are shown in Figure 9, and the corresponding discretized NMSE and the summed relative errors are summarized in Table 7. The chosen lateral ranges of three times the local spot sizes caused all amplitude shapes to appear identical. This was also the case for the shapes of the phase profiles. However, this was not immediately visible due to phase wrapping, particularly in the far and extremely far fields, which could be resolved by using a phase-tracking algorithm. Figure 10 shows the unwrapped phase for a propagation distance of about 3 km, i.e., 1000 $z_{r}$.

In Figure 9, it can clearly be seen that both the MEM and GBD accurately represent the circular Gaussian beam. However, for all shown propagation distances, the MEM is more accurate than the GBD (column on the right-hand side in the graphs), which is also reflected in the discretized NMSE $\varepsilon^{\mathrm{DNMSE}}$ (column 3) and summed relative error $\varepsilon_{\sum }^{\mathrm{rel}}$ (column 4) in Table 7. The DNMSE error of the MEM is notably small, considering that the waist of the modes used in the MEM determined using Equation (19) was 0.8 mm, which closely aligns with the waist of the non-clipped Gaussian beam. While it is possible to obtain an error of 0 by choosing the waist of the modes equal to the non-clipped beam waist of 1 mm, such a comparison would be meaningless in this particular scenario. However, this agrees with the analytically calculated NMSE (Equation (12)), which was 1.3989 $\times \;10^{-14}$. The discretized NMSE of the MEM result was found to be slightly smaller than this, with a residual propagation distance dependency. Both of these properties originate from the finite radial range used in the decomposition and error computation.

In conclusion, we find that in this example, both methods accurately resolved the incident wavefront. The MEM exhibited exceptional precision and was, therefore, more accurate compared to the GBD.

A particular challenge in this simulation was the ultra-large propagation distance of 3 million kilometers. This naturally gave rise to numerical precision problems. Additionally, it was a major concern and one of the initial reasons for why we tested this extremely far field, which is relevant for space-based gravitational wave detectors. The given example shows no apparent signs of numerical limitations. This was achieved by a separation of the optical pathlength (i.e., the ikz-term in the Gaussian beam), from the residual phase contributions [50].

Additionally, it was expected by our community that the GBD could not propagate the beam into this extremely far field without a re-decomposition in an intermediate plane. This expectation originated from the chosen small grid in the original decomposition plane compared to the very large spot size in the target plane. The concern was that a combination of small and large numbers would result in limitations due to numerical precision. After all, in the given example, the spot size of the total beam was 1 mm in the decomposition plane, for which we chose a window size of 8 mm. Since the beam was not re-decomposed, this was also used in the target plane, where the Gaussian beam radius increased to 1.0160 $\times \;10^{3}$ km (cf. Figure 9). Yet, despite the grid being unfit for the target plane, it is clearly visible in Figure 9 that the beam was well represented. A re-decomposition was not needed in the given example.

##### General Astigmatic Gaussian Beam

**Figure 11:**
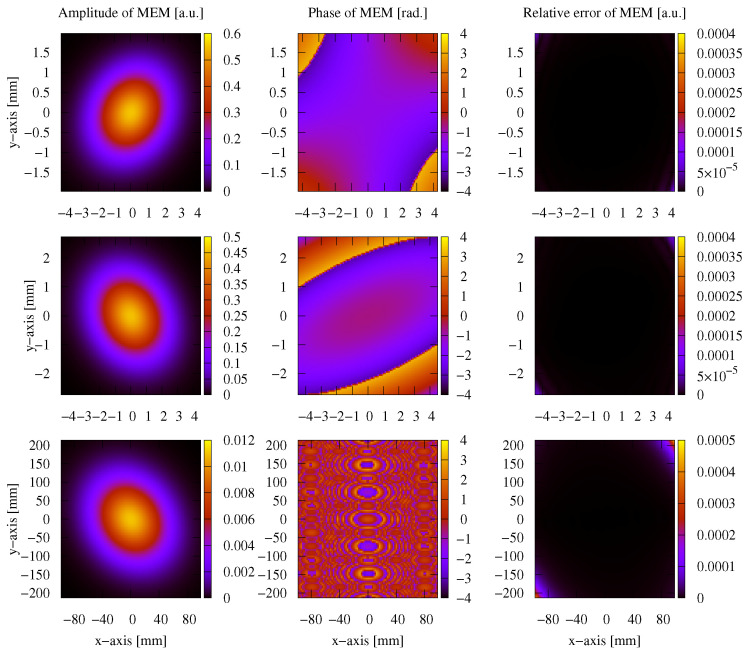
MEM representation of a general astigmatic Gaussian beam at different propagation distances. From left to right: amplitude (absolute value), phase, and relative error distributions of the MEM. From top to bottom: propagation distances of 29.526 mm, 2.9526m, and 295.26m. All the simulation parameters are listed in Table 8. It can be seen in the third column of this figure that the MEM demonstrated good agreement with the initial general astigmatic Gaussian beam.

**Figure 12:**
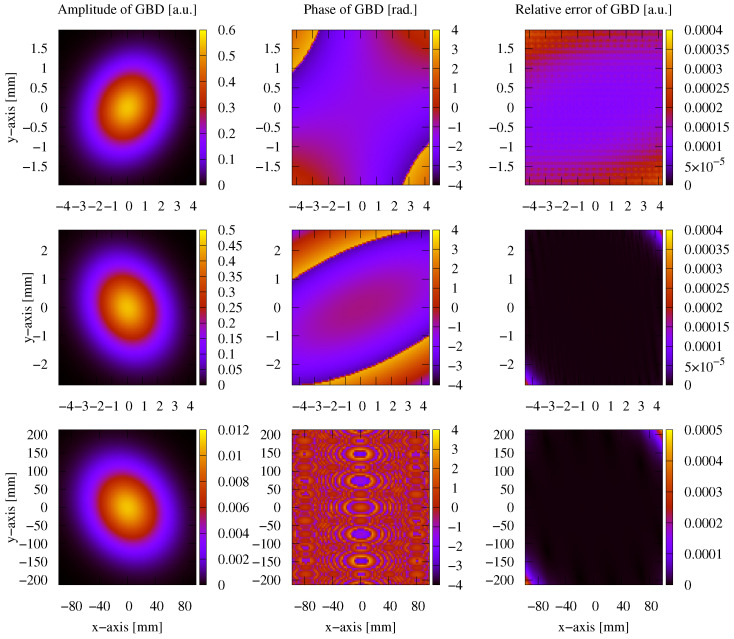
GBD representation of a general astigmatic Gaussian beam at different distances. From left to right: amplitude (absolute value), phase, and relative error distributions of the GBD. From top to bottom: propagation distances of 29.526 mm, 2.9526m, and 295.26m. All the simulation parameters are listed in Table 8. It can be seen in the third column of this figure that the GBD demonstrated good agreement with the initial general astigmatic Gaussian beam.

After testing the performance in the case of a non-clipped circular symmetric Gaussian beam, we now test the performance of these methods on a general astigmatic Gaussian beam. In this case, two beam waists and a complex angular orientation θ need to be defined [51]. The parameters used for this performance comparison are listed in Table 8. In this case, the lateral parameter *y* was not set to 0. As shown in Section 3, we used a mode order of N=50 and compared this with a GBD grid size of 300×300. The resulting electric field profiles of the MEM were plotted, as shown in Figure 11, as were those of the GBD, as shown in Figure 12, for three different propagation distances of $z_{r1}/100,z_{r1},$ and $100z_{r1}$, with $z_{r1}=2.9526m$ being the XZ-plane Rayleigh range. We do not present the results for 3 Gm in this case because the sampling points of 101×101 were too low for such an extremely far distance, and we have already demonstrated the accuracy of both the MEM and GBD at 3 Gm for the non-clipped circular Gaussian beam. We computed the electric field in lateral distances (x,y) up to twice the spot size. This provided good visibility of the amplitude profiles; however, it slightly masked the magnitude of ellipticity in the resulting images.

**Figure 13:**
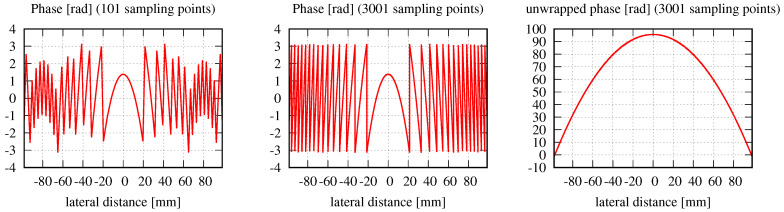
Cross-section of the phase obtained using the MEM along y=0 and z=295.26m. The image on the left shows the phase using 101 sampling points, the image in the center shows the phase using 3001 sampling points, and the image on the right shows the phase using 3001 sampling points after a phase-tracking algorithm unwrapped the data. One can see that the small side maxima, which are seen as circles in Figure 11 and Figure 12, disappear when a higher sampling rate is used, and indeed a smooth but strongly curved phase profile is restored in the far field.

In the first column of both figures, one can see the well-known characteristic of a general astigmatic Gaussian beam: its elliptical amplitude pattern, which undergoes a rotating orientation during propagation. Both methods equally captured this characteristic. The phase of the general astigmatic beam, as shown in the second column, is mostly smooth at propagation distances less than 3 m (top and central row), except for two lines of phase jumps. In the far field (lowest row), there is a pattern of circular shapes. This pattern is an aliasing effect resulting from the high wavefront curvature, and the consequent high number of phase jumps in combination with the low sampling rate. We demonstrate this effect and how it can be resolved for a cross-section of the phase profile in Figure 13. Finally, the third column of Figure 11 and Figure 12 shows the accuracy of each method. The DNMSE and summed relative error of the MEM and GBD at different propagation distances are shown in Table 9.

It can be seen in this table, as well as in the third column of both Figure 11 and Figure 12, that the MEM was again, consistently more accurate compared to the GBD. Additionally, one can see that both the DNMSE and summed relative error of the MEM increased with the propagation distances, whereas for the GBD, the DNMSE nonmonotonically decreased with the propagation distances, and the summed relative error again changed inconsistently.

#### 4.1.2. Clipped Gaussian Beam

In this subsection, we directly compare the performance of the MEM and GBD for a circular Gaussian beam clipped by a circular aperture. For convenience, the parameter settings are summarized in Table 10.

**Figure 14:**
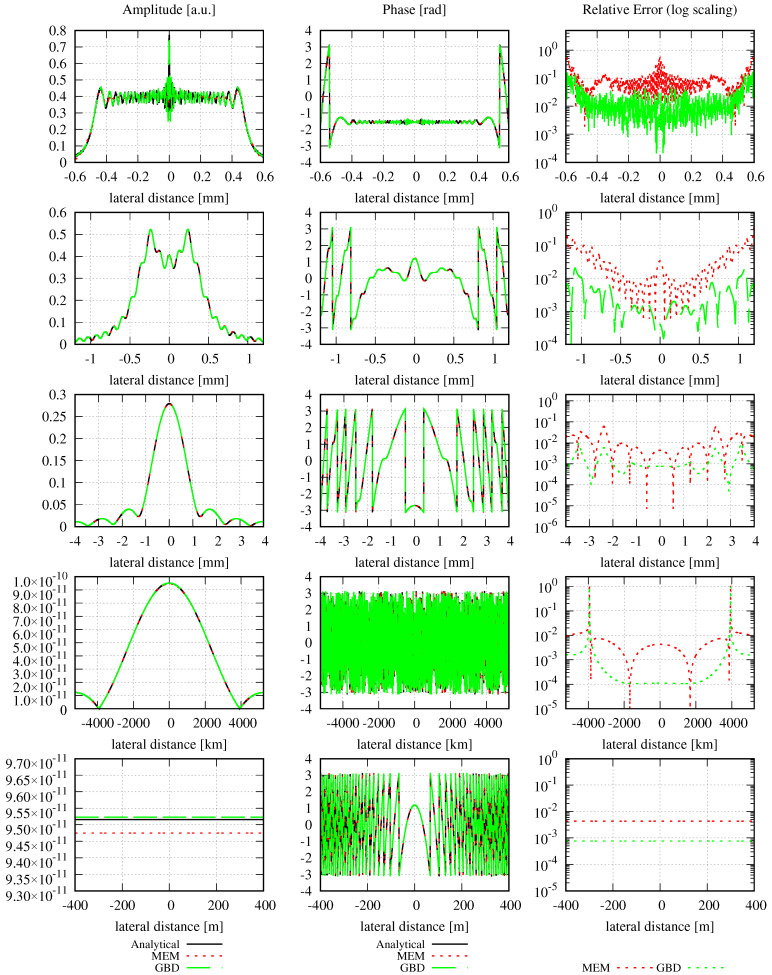
Amplitude (absolute value), phase, and relative error distributions of a clipped circular Gaussian beam with propagation distances of 5 mm and 100 mm in the near field, 1000 mm in the far field, and 3 Gm in the extremely far field (from top to bottom). The lateral range in the fourth row is twice the local spot size. The simulation parameters are listed in Table 10. The analytical methods used for the near and far fields were those of Campbell [46] and Tanaka et al. [47], respectively. The graphs in the third column show the relative error, which indicates that the GBD performed better than the MEM.

In this case, the diffracted beam propagated 5 mm and 100 mm in the near field, 1000 mm in the far field, and 3 Gm in the extremely far field, with Fresnel numbers of 46.9925, 2.3496, 0.2350, and $7.83208\times 10^{-11}$, respectively. The resulting amplitude, phase, and relative error profiles are depicted in Figure 14, and the corresponding errors are summarized in Table 11. We chose lateral ranges that allowed for good visibility of the amplitude profiles. For 3 Gm, we provided two lateral ranges: approximately twice the spot size and a smaller lateral range of 400 m. In fact, we did not attempt to compute the exact spot size of the clipped Gaussian beam but simply estimated it from two boundary cases: the spot sizes of a top hat beam and a Gaussian beam. The spot size of a top hat beam with a radius of 0.5 mm can be estimated using Equation (8) in [48], resulting in 2625.3 km after 3 Gm. For a Gaussian beam with a waist of 0.5 mm, the spot size at a propagation distance of 3 Gm would be 2032.1 km, and the spot size of the clipped Gaussian beam is expected to be between these two values. Here, for simplicity, we used the spot size of the top-hat beam for the lateral ranges.

In Figure 14, it can be seen that both methods effectively described the clipped Gaussian beam; however, in all the shown propagation distances, the GBD behaved better compared to the MEM. Quantitatively, we can also draw the same conclusion based on the discretized NMSE and summed relative errors listed in Table 11. Particularly in the near field with propagation distances of 5 mm and 100 mm, the differences in the errors of the MEM and GBD were significant. This aligns with the findings in Section 2.1.4, which showed that the MEM insufficiently resolved the high-frequency spatial oscillation in the very near field. With the increase in the propagation distance, at 1000 (third row), the differences between both methods became smaller, and in the extremely far field 3 Gm (fourth and lowest row), they narrowed even further. We can also see that both methods became more accurate with increasing propagation distances. As before, the extremely far electric field was computed by the GBD in one step and did not require a re-decomposition in an intermediate plane.

**Figure 15:**
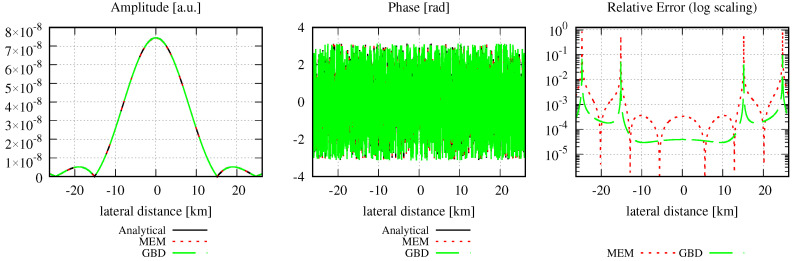
Amplitude (absolute value), phase, and relative error distributions of a clipped circular Gaussian beam at propagation distances of 3 Gm. Both the beam waist diameter and aperture diameter are 30 cm. The image on the right shows the relative error, indicating that the GBD performed better than the MEM.

An aperture radius of 0.5 mm is a typical value used in laboratory experiments and, therefore, fits well with the shown near-field propagation distances. However, it is not a realistic value for the extremely far-field simulation case originating from space-gravitational wave detectors. The aperture diameter in LISA-like missions is usually between 20 cm and 40 cm. Therefore, here, we illustrate another example, where both the beam waist diameter and aperture diameter are 30 cm for a propagation distance of 3 Gm. Other parameters are chosen from Table 10, except for the window size, which is 35 cm in this case, and the lateral range was chosen to be 3 times the spot size estimated using Equation (8) in [48]. The resulting amplitude, phase, and relative errors are shown in Figure 15.

In this figure, it can be seen that the order of magnitude of the amplitude was $10^{-8}$ (image on the left), and the relative error shown in the image on the right indicates that the GBD was again more accurate compared to the MEM.

### 4.2. Aberrated Wavefronts

An aberration is a typical phenomenon in optics and results in a case where the beam cannot be propagated analytically through the setup. For instance, in LISA-like missions, an aberration occurs when the beam propagates through the telescope. The beam that is launched toward the remote spacecraft is, therefore, not a perfectly clipped Gaussian beam but has additional wavefront distortions. Likewise, the received beam is not a perfect top-hat beam. These aberrations can affect the readout noise and, therefore, need to be studied.

Here, we study how the MEM and GBD decompose and propagate aberrated wavefronts. Unfortunately, we do not have an analytic solution to compare the results with. We can, therefore, only qualitatively compare the results of both methods without being able to directly assess which one is more accurate. Instead, we test whether the methods generate qualitatively agreeable results.

Mathematically, a wavefront aberration can be described by adding an additional phase term $\Omega_{a}$ in the complex electric field of the original beam, i.e.,

(38) $$E_{a}\left(r;0\right)=E\left(r;0\right)\exp\left(ik\Omega_{a}\right)\;,$$

where $E_{a}$ is the complex electric field of the beam with aberration, *E* is the complex electric field of the original beam, and $\Omega_{a}$ is the additional phase distribution caused by the aberration. Such a phase term is usually described using Zernike polynomials [52]:

(39) $$\Omega_{a}\left(x,y\right)=\sum_{n=0}^{N}\sum_{m=-n}^{n}c_{n}^{m}\;Z_{n}^{m}\left(x,y\right),$$

where $c_{n}^{m}$ represents the coefficients and $Z_{n}^{m}$ are the Zernike polynomials, where n−m≥0 is an even number.

To demonstrate that both the MEM and GBD can describe wavefront aberrations, we present the results for the well-known effects caused by individual Zernike polynomials up to the fourth order (cf. Figure 4 in [53], Figure 3 in [54], and Figure 9.8 in [55]). The impact of optical aberrations is often represented by calculating the point-spread function (PSF) [53], i.e., the intensity profile at a distance *z*, which is usually computed through Fourier transformation:

(40) $$\mathrm{PSF}\left(r;z\right)=|{\mathrm{FT}}_{z}\left(E_{a}\left(r;0\right)\right){|}^{2}\;,$$

which is also known as the response of the pupil function after Fourier transform (FT) at a certain distance *z* [56]. In the far field, the beam source can usually be regarded as a point source compared to the propagation distance, and the PSF equals the intensity profile computed by Fraunhofer diffraction. In this section, we use the MEM and GBD to represent the effect of optical aberrations by calculating the amplitude profile of a diffracted Gaussian beam with aberrations at the Fraunhofer region, rather than directly calculating the PSF.

In this example, we calculated the amplitude profiles of the first four orders of Zernike polynomials using the MEM and GBD for an aperture with a radius of 1 mm at λ=1064nm, and the coefficients of the Zernike polynomials $c_{n}^{m}$ for all *m* and *n* were set to 10. The parameter settings for this example are listed in Table 12. The propagation distance was 5 km ($F=1.8797\times 10^{-4}$) and, therefore, in the Fraunhofer region.

**Figure 16:**
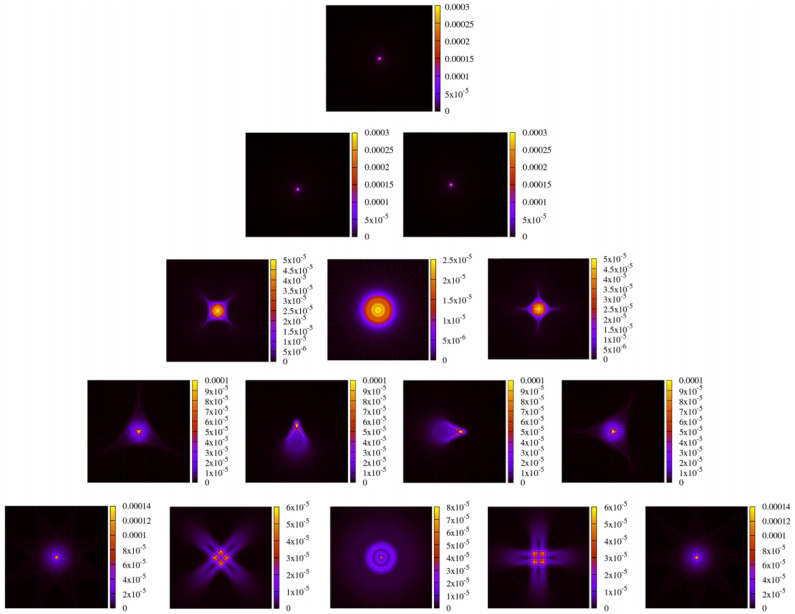
Amplitude profile for the first 4 orders of wavefront aberrations described by Zernike polynomials, generated using the GBD method. Aberrations of order n = 1 are called tilt; n = 2 are called astigmatism and defocus; n = 3 are called coma and trefoil; and n = 4 are called tetrafoil, 2nd astigmatism, and spherical aberration. The images each cover 200 m × 200 m each.

For a good resolution of our results, we used 201×201 = 40,401 sampling points. For the MEM, we chose a mode order of N=50, and for the GBD, we used a grid size of G=150×150. The results computed using the GBD are shown in Figure 16, showing the expected amplitude profiles (compare e.g., (cf. Figure 4 in [53], Figure 3 in [54], and Figure 9.8 in [55]).

**Figure 17:**
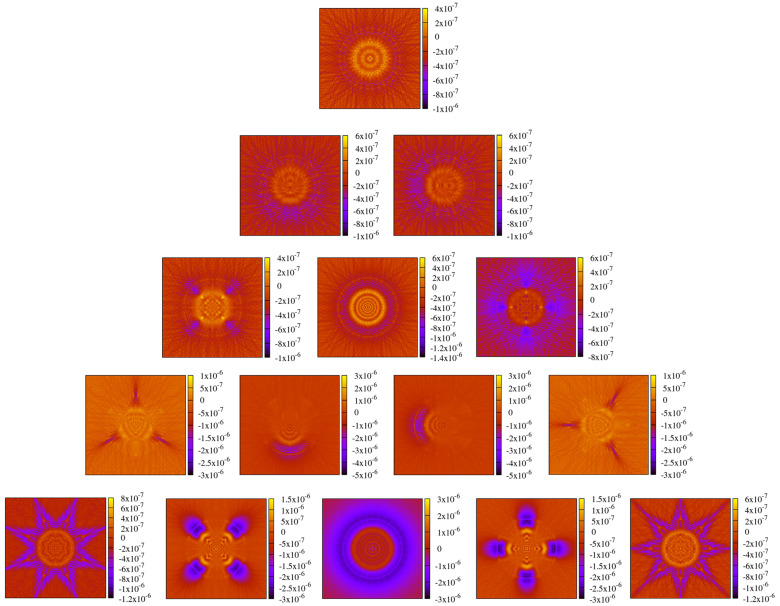
Differences between the amplitude profiles of the MEM and GBD for the first 4 orders of wavefront aberrations described by Zernike polynomials. The images each cover 200 m × 200 m.

The MEM results were almost identical and are not shown here to avoid unnecessary duplication. Instead, we illustrate the difference between the MEM and GBD results in Figure 17. It can be seen in this figure that the difference between the MEM and GBD was in the order of $10^{-7}$ to $10^{-6}$. If this was related to the individual amplitudes of approximately $10^{-5}$ to $10^{-4}$, we would speak of relative deviations in the order of a few percent. Even though we cannot assess which of the two methods was more accurate, we can conclude that generally, either method can be used for decomposing and propagating aberrated wavefronts. Please note that we intentionally only plotted here the difference between the results and performed a qualitative relative deviation because the computation of a relative difference like $|E_{\mathrm{GBD}}-E_{\mathrm{MEM}}\left|/\right|E_{\mathrm{MEM}}|$ caused divisions by zero with the shown lateral ranges.

### 4.3. Reflection from Optical Components

**Figure 18:**
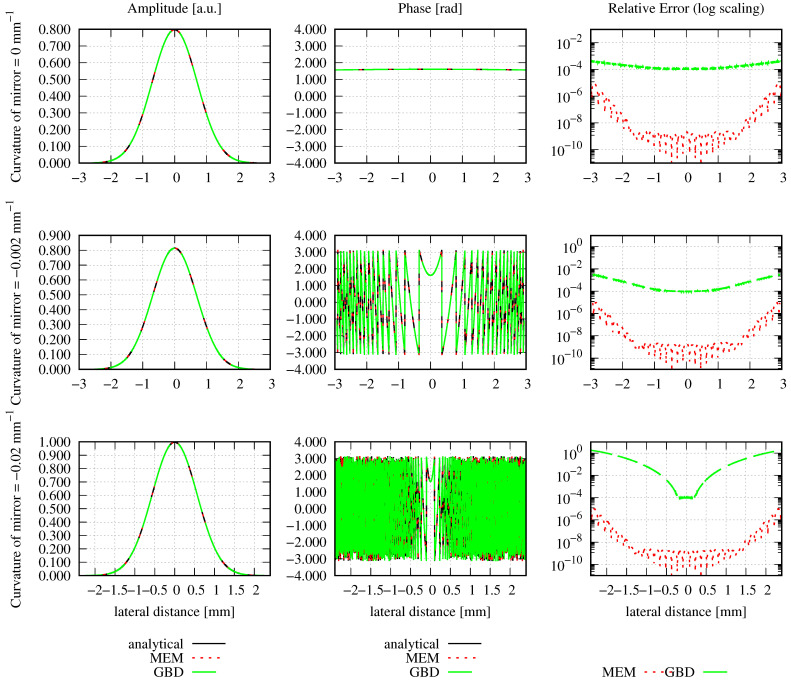
Amplitude, phase distribution, and relative error after the beam was reflected from mirrors with different curvatures. The graphs in each row represent the amplitude, phase, and relative errors, respectively, from left to right, and each row represents a different curvature of the mirrors of 0 mm${}^{-1}$, −0.002 mm${}^{-1}$, and −0.02 mm${}^{-1}$ from top to bottom. The simulation parameters are listed in Table 13. The relative deviation of the GBD method from the Gaussian beam and the MEM increased as the curvature of the mirror increased.

**Figure 19:**
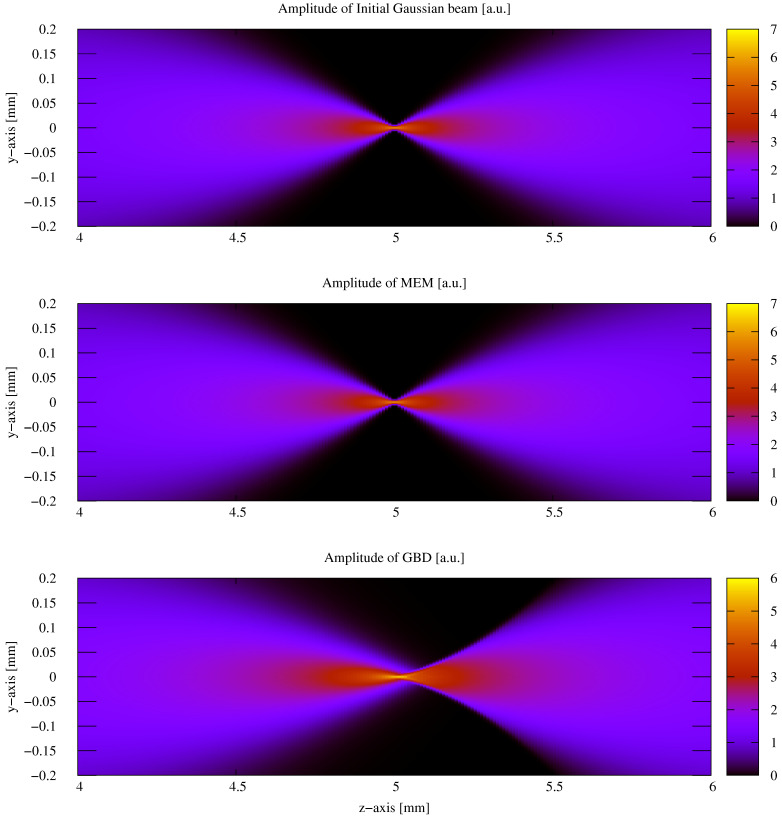
Amplitude profile of the initial Gaussian beam, the MEM, and the GBD through longitudinal sections near the focal point after being reflected from a concaved spherical mirror with a curvature of −0.1 mm${}^{-1}$. The amplitude was scaled by log(Amplitude + 1). Only the GBD showed signs of spherical aberrations.

In the previous subsections, the behavior of the MEM and GBD in free space propagation was compared. However, a major difference between both methods arose when the decomposed beams were propagated through an optical setup. This is an important test case because of the differences in the decomposition methods: while all MEM modes share the same axis, the GBD grid beams are distributed on a grid. To compute the interaction of a beam with a surface, the usual implementations, especially the implementation in IfoCAD, compute the second fundamental form of the surface at the intersection point. Consequently, when an MEM beam reflects or refracts, it effectively interacts only with the intersection point and its local curvature, whereas the GBD beam probes the surface at multiple intersection points. We illustrate this difference with a simple test case: the reflection of a Gaussian beam from a spherically curved mirror with varying curvatures. We expected the GBD method to show increasing levels of spherical aberration with increasing mirror curvature, whereas the MEM was not expected to resolve the occurring spherical aberrations. This means that we decomposed a Gaussian beam with the MEM and GBD and reflected the original Gaussian, as well as the MEM and GBD representations of this Gaussian, from a spherically curved mirror. We then expected an increasing level of deviation between the GBD beam and the reference Gaussian for increasing mirror curvatures, whereas the MEM was not expected to show this behavior. The results of this simple test are illustrated in Figure 18, and the results for a stronger curvature case are shown in Figure 19, confirming the expected behavior.

For this simulation, we assumed a Gaussian beam with a 1 mm waist radius located at its origin, propagated by z=10mm before it impinged orthogonally and centered onto the spherically curved mirror, which had a diameter of x mm. The mirror was, therefore, sufficiently oversized to reflect the full beam. After reflection, the complex electric field was calculated at an observation plane, which was 5mm away from the mirror and orthogonal to the beam. All simulation parameters are summarized in Table 13.

Please note that we use the term ‘relative error’ in Figure 18 for consistency with the previous sections. However, this should be seen as a relative deviation, given that the Gaussian beam, which served as a reference, could not be trusted to be physically correct in this example because it was insensitive to spherical aberrations.

## 5. Summary and Conclusions

In this paper, we have compared two wavefront decomposition methods—the MEM and GBD—for different test cases. To assess the performance of the methods and allow a direct comparison of both, several different types of error estimates were introduced: the normalized mean-square error (NMSE), its discrete analog counterpart (DNMSE), and the relative error and its sum. The properties of all these errors were discussed and compared.

For the MEM, we found that even though the well-known NMSE is propagation distance-independent, the DNMSE is usually not because the lateral ranges chosen in the target plane are too small. To achieve the propagation independence of the DNMSE, the lateral ranges in the given example were so large that the MEM with mode orders up to 50 was unable to resolve the necessary lateral range due to the finite spot sizes of the involved modes.

While the NMSE and its discretized analog counterpart are commonly known and used errors, they do not enable visualizing the error distribution over the cross-section of the field of interest. For this, the relative error can be used. Finally, the summed relative error is a useful addition to the relative error, providing quantification of the graphical findings.

To perform a direct comparison, we tested the simulation runtime for different settings in a typical test case. We showed that the runtime of the GBD heavily depends on the number of sampling points in the target plane, unlike the MEM. Therefore, a direct comparison of the precision of these methods for comparable runtimes heavily depends on the number of sampling points chosen in the target plane. Naturally, this finding depends on the chosen software tool and the implementation of the methods. All simulations in this paper were performed using the software library IfoCAD (runtime tests with version 2022/10, git commit adf19a5b). However, the introduced method of determining settings that facilitate a fair comparison remains independent of computer systems, software choices, and implementation details. Additionally, these findings underline that in any software implementation, the GBD method should not only be optimized for the decomposition but also for the superposition in the target plane.

For the individual performance of the MEM, we found that it does not ideally resolve the high-frequency spatial oscillations in the near field but accurately resolves the far-field wavefronts, even with small mode orders. The accuracy of the MEM naturally improves with higher mode orders, but also with the usual ranges of interest and propagation distance.

For the individual performance of the GBD method, we likewise found that the high-frequency spatial oscillations in the near field are insufficiently resolved with the typical settings. The comparably smooth far fields, in comparison, are resolved with higher accuracy. Naturally, the accuracy of the GBD method improves with increasing grid sizes. However, this can quickly result in grid beam waists that are so small that they violate the paraxial approximation. We intentionally tested the performance of the GBD method in such an imperfect case and found that the precision was not impaired by the non-ideal, extremely small waist sizes. This means that the relative error and its sum decreased for GBDs with increasing grid sizes, despite the use of increasingly smaller waist sizes that violated the paraxial approximation.

We directly compared the MEM and GBD for cases where the electric field’s amplitude and phase were analytically known in different propagation distances. We performed this test for typical propagation distances in the near and far fields, as well as in the extremely far field spanning millions of kilometers, as needed for space-gravitational wave detectors. We showed that both methods can resolve the field at this extremely far distance without the need for a re-decomposition at an intermediate distance. The direct method comparison showed the superior performance of the MEM for the decomposition and free-beam propagation of non-clipped circular and general astigmatic Gaussian beams. In the cases of clipped circular Gaussian beams, the GBD method exhibited higher accuracy. However, these findings may depend on the settings and the software used to implement both methods, as well as the computer, operation system, and compilers utilized.

Both methods (the MEM and GBD) performed decompositions into beams derived under the paraxial approximation assumption. Therefore, it is rather significant that the limited accuracy we described for the MEM in the near field behind an aperture matched a case where the paraxial approximation did not apply. In this specific case, diffraction caused beam elements with a high angle to the principal optical axis to contribute significantly to the overall wavefront. In comparison, the GBD achieved a higher accuracy and may be better suited for resolving the field closely behind an aperture. This is because the grid beams are distributed over a given surface, resulting in smaller angles relative to the grid beam axes.

Additionally, we compared the MEM and GBD representations of aberration and showed a qualitative agreement between the results. Finally, in one example, we illustrated that the GBD method is a superior method for propagation through an optical setup, especially when interactions with surfaces occur. While the MEM decomposed the initial field into modes that all share the same beam axis, the GBD method decomposed it into fundamental Gaussian beams on a grid. Consequently, the MEM beam probed the curvature of a surface only at one intersection point between its beam axis and the surface, whereas the GBD grid beams probed the surface curvature at multiple intersection points. We demonstrated this difference through a qualitative comparison of the electric fields of a Gaussian beam and its MEM and GBD representations after reflection from a curved mirror. Moreover, we showed that the GBD beam exhibited the expected spherical aberration, unlike the Gaussian or MEM beams.

We can generally conclude that both methods are useful for decomposing and propagating non-Gaussian beams. Once the fields are decomposed, propagation in free space or through an optical setup is computationally trivial. The method that proves more accurate depends on the specific test case and simulation settings. However, for propagation through optical layouts, especially when the beam interacts with surfaces, and particularly if non-spherical surfaces exist in the setup, the GBD method, with its grid of beams, holds a clear advantage over the MEM.

## Figures and Tables

**Table 1:** Parameter list for the MEM example.

Parameter	Description	Value
λ	wavelength	1064 nm
$P_{0}$	beam power	1 W
$w_{0}$	beam waist	2 mm
$z_{0}$	distance from the waist	0 mm
$R_{a}$	aperture radius	0.5 mm
*N*	mode order of the MEM	10, 20, 30, 40, 50
$w_{0d}$	waist of the modes used in the MEM	0.2236 mm, 0.1581 mm, 0.1291 mm, 0.1118 mm, 0.1 mm
*d*	propagation distance	5 mm, 20 mm, 100 mm, 1000 mm
*X*	number of sampling points	3001

**Table 2:** The MEM errors, including the NMSE, discretized NMSE, and summed relative error, defined in Equation (12), Equation (13), and Equation (18), respectively, are calculated for increasing mode orders at different propagation distances. The NMSE and discretized NMSE, which are propagation distance-independent, are numerically equivalent when the lateral ranges *R* are large enough. For smaller lateral ranges *R*, the discretized NMSE is propagation distance-dependent. The summed relative error decreases with increasing mode order for any propagation distance, and for a given mode order, it increases (but not consistently) with increasing propagation distance. The number of sampling points *X* is 3001.

Propagation Distance (mm)	Mode Order	NMSE $\varepsilon^{\mathrm{NMSE}}\left(N,w_{0d}\right)$	DNMSE (for Larger Lateral Ranges) $\varepsilon_{\circ }^{\mathrm{DNMSE}}\left(N_{R},R,z\right)$	DNMSE (for Smaller Lateral Ranges) $\varepsilon_{\circ }^{\mathrm{DNMSE}}\left(N_{R},R,z\right)$	The Summed Relative Error (for Larger Lateral Ranges) $\varepsilon_{\sum }^{\mathrm{rel}}\left(N_{R},R,z\right)$	The Summed Relative Error (for Smaller Lateral Ranges) $\varepsilon_{\sum }^{\mathrm{rel}}\left(N_{R},R,z\right)$
	10	0.0527	0.0519	0.0436	13.6	0.964
	20	0.0275	0.0268	0.021	12.8	0.731
5	30	0.0186	0.0178	0.0116	12.5	0.486
	40	0.0139	0.0132	0.0077	12.3	0.344
	50	0.0112	0.0105	0.0057	12.2	0.247
	10	0.0527	0.0517	0.0444	99.1	2.54
	20	0.0275	0.0265	0.0192	98.3	1.75
20	30	0.0186	0.0175	0.0101	97.7	1.12
	40	0.0139	0.013	0.0057	97.1	0.535
	50	0.0112	0.0102	0.0042	96.5	0.463
	10	0.0527	0.0517	0.0323	2.51 $\times \;10^{3}$	5.11
	20	0.0275	0.0265	0.0083	2.51 $\times \;10^{3}$	2.46
100	30	0.0186	0.0175	0.0035	2.5 $\times \;10^{3}$	2.28
	40	0.0139	0.013	$8.85\times 10^{-4}$	2.5 $\times \;10^{3}$	1.22
	50	0.0112	0.0103	$1.95\times 10^{-4}$	2.49 $\times \;10^{3}$	0.538
	10	0.0527	0.0516	0.0051	2.04 $\times \;10^{5}$	28.4
	20	0.0275	0.0264	$6.08\times 10^{-4}$	2.03 $\times \;10^{5}$	8.48
1000	30	0.0186	0.0174	$1.86\times 10^{-4}$	2.03 $\times \;10^{5}$	4.56
	40	0.0139	0.0129	$6.94\times 10^{-5}$	2.03 $\times \;10^{5}$	2.77
	50	0.0112	0.0102	$2.57\times 10^{-5}$	2.02 $\times \;10^{5}$	1.68

**Table 3:** Parameter list for the GBD example.

Parameter	Description	Value
λ	wavelength	1064 nm
$P_{0}$	beam power	1 W
$w_{0}$	beam waist	2 mm
$z_{0}$	distance from the waist	0
$R_{a}$	aperture radius	0.5 mm
*G*	grid size of the GBD	100×100, 200×200, 500×500, 1000×1000
*L*	window size of the GBD	1.5 mm
$f_{\mathrm{ws}}$	waist scaling factor of the GBD	1.5
$w_{0g}$	grid beam waist of the GBD	11.2 μm, 5.6 μm, 2.2 μm, 1.1 μm
grid shape	grid shape of the GBD	square
*d*	propagation distance	5 mm, 20 mm, 100 mm, 1000 mm
*X*	the number of sampling points	3001

**Table 4:** The GBD errors, including the discretized NMSE and the summed relative error, defined in Equation (32) and Equation (37), respectively, are calculated for increasing grid sizes at different propagation distances. The discretized NMSE for both lateral ranges are propagation distance-dependent. The summed relative errors for smaller, lateral ranges decrease with increasing grid sizes at any propagation distance. The number of sampling points *X* is 3001.

Propagation Distance (mm)	Grid Size	DNMSE (for Larger Ranges) $\varepsilon_{\circ }^{\mathrm{DNMSE}}\left(N_{R},R,z\right)$	DNMSE (for Smaller Ranges) $\varepsilon_{\circ }^{\mathrm{DNMSE}}\left(N_{R},R,z\right)$	The Summed Relative Error (for Larger Ranges) $\varepsilon_{\sum }^{\mathrm{rel}}\left(N_{R},R,z\right)$	The Summed Relative Error (for Smaller Ranges)$\varepsilon_{\sum }^{\mathrm{rel}}\left(N_{R},R,z\right)$
5	100×100	0.0158	0.0134	11.83	0.471
	200×200	0.0196	2.57 $\times \;10^{-3}$	15.7	0.189
	500×500	1.95 $\times \;10^{-3}$	5.84 $\times \;10^{-4}$	12.0	0.0886
	1000×1000	3.69 $\times \;10^{-4}$	5.84 $\times \;10^{-5}$	5.72	0.0304
20	100×100	0.0126	6.78 $\times \;10^{-3}$	96.2	0.967
	200×200	5.38 $\times \;10^{-3}$	8.57 $\times \;10^{-4}$	97.6	0.381
	500×500	1.20 $\times \;10^{-4}$	6.92 $\times \;10^{-5}$	81.81	0.111
	1000×1000	1.93 $\times \;10^{-5}$	5.98 $\times \;10^{-6}$	40.54	0.0253
100	100×100	9.80 $\times \;10^{-3}$	8.39 $\times \;10^{-4}$	2.49 $\times \;10^{3}$	1.15
	200×200	5.26 $\times \;10^{-3}$	9.53 $\times \;10^{-5}$	2.59 $\times \;10^{3}$	0.273
	500×500	1.22 $\times \;10^{-3}$	1.50 $\times \;10^{-6}$	2.89 $\times \;10^{3}$	0.040
	1000×1000	1.94 $\times \;10^{-4}$	6.82 $\times \;10^{-7}$	1.45 $\times \;10^{3}$	0.0251
1000	100×100	9.87 $\times \;10^{-3}$	4.64 $\times \;10^{-5}$	2.08 $\times \;10^{5}$	2.38
	200×200	5.14 $\times \;10^{-3}$	8.25 $\times \;10^{-6}$	2.32 $\times \;10^{5}$	1.55
	500×500	1.10 $\times \;10^{-3}$	1.40 $\times \;10^{-7}$	3.33 $\times \;10^{5}$	0.141
	1000×1000	1.47 $\times \;10^{-4}$	7.55 $\times \;10^{-8}$	1.37 $\times \;10^{5}$	0.0780

**Table 5:** The DNMSE and the summed relative error for different grid shapes at different propagation distances, given a grid size of 500×500. It can be seen that the hexagonal grid caused smaller errors for short propagation distances of 5 mm and 20 mm, whereas the square grid generated more precise results at higher propagation distances. The number of sampling points *X* is 3001.

Propagation Distance (mm)	Grid Shape	DNMSE $\varepsilon_{\circ }^{\mathrm{DNMSE}}\left(N_{R},R,z\right)$	The Summed Relative Error $\varepsilon_{\sum }^{\mathrm{rel}}\left(N_{R},R,z\right)$
5	square	5.84 $\;\times \;10^{-4}$	0.0886
	hexagonal	2.41 $\;\times \;10^{-4}$	0.0603
20	square	6.92 $\;\times \;10^{-5}$	0.111
	hexagonal	3.12 $\;\times \;10^{-5}$	0.0641
100	square	1.50 $\;\times \;10^{-6}$	0.0396
	hexagonal	3.82 $\;\times \;10^{-6}$	0.0583
1000	square	1.40 $\;\times \;10^{-7}$	0.141
	hexagonal	2.50 $\;\times \;10^{-7}$	0.167

**Table 6:** Parameter list for non-clipped circular Gaussian beam.

Parameter	Description	Value
λ	wavelength	1064 nm
$P_{0}$	beam power	1 W
$w_{0}$	beam waist	1 mm
$z_{0}$	distance from the waist	0 mm
$R_{a}$	aperture radius	4 mm
*N*	mode order of the MEM	50
$w_{0d}$	waist of the modes used in the MEM	0.8 mm
*G*	grid size of the GBD	400 × 400
*L*	window size of the GBD	8 mm
$f_{\mathrm{ws}}$	waist scaling factor of the GBD	10/3
$w_{0g}$	grid beam waist of the GBD	0.0333 mm
grid shape	grid shape of the GBD	square
*d*	propagation distance	$0.001z_{r}$, $z_{r}$, $1000z_{r}$, 3 Gm
*X*	number of sampling points	3001

**Table 7:** The discretized NMSE and the summed relative error of the MEM and GBD, respectively, for different propagation distances. The number of sampling points *X* is 3001.

Propagation Distance	Method	DNMSE $\varepsilon_{\circ }^{\mathrm{DNMSE}}$	The Summed Relative Error $\varepsilon_{\sum }^{\mathrm{rel}}$
2.95 mm	MEM	1.31 $\times \;10^{-17}$	5.00 $\times \;10^{-5}$
	GBD	1.38 $\times \;10^{-8}$	7.06 $\times \;10^{-3}$
2.95 m	MEM	4.16 $\times \;10^{-15}$	0.0011
	GBD	1.02 $\times \;10^{-11}$	0.0019
2.95 km	MEM	5.11 $\times \;10^{-15}$	371
	GBD	1.02 $\times \;10^{-11}$	518
3 Gm	MEM	1.35 $\times \;10^{-14}$	3.89 $\times \;10^{14}$
	GBD	1.02 $\times \;10^{-11}$	2.23 $\times \;10^{15}$

**Table 8:** Parameter list for non-clipped general astigmatic Gaussian beam.

Parameter	Description	Value
λ	wavelength	1064 nm
$P_{0}$	beam power	1 W
$w_{01}$	beam waist in XZ plane	1 mm
$z_{01}$	distance from the waist in XZ plane	0 mm
$w_{02}$	beam waist in YZ plane	2 mm
$z_{02}$	distance from the waist in YZ plane	0 mm
θ	tilt angle	0.1 + 0.2i
$R_{a}$	aperture radius	8 mm
*N*	mode order of the MEM	50
$w_{0d}$	waist of the modes used in the MEM	1.6 mm
*G*	grid size of the GBD	300 × 300
*L*	window size of the GBD	16 mm
$f_{\mathrm{ws}}$	waist scaling factor of the GBD	10/3
$w_{0g}$	grid beam waist of the GBD	0.0444 mm
grid shape	grid shape of the GBD	square
*d*	propagation distance	$0.01\;z_{r1}$, $z_{r1}$, $100\;z_{r1}$
*X*	number of sampling points	101×101=10,201

**Table 9:** The DNMSE and summed relative error of the MEM and GBD for the non-clipped general astigmatic Gaussian beam at different propagation distances. The number of sampling points *X* is 10201.

Propagation Distance	Method	DNMSE $\varepsilon_{□}^{\mathrm{DNMSE}}$	The Summed Relative Error $\varepsilon_{\sum }^{\mathrm{rel}}$
29.5 mm	MEM	6.25 $\times \;10^{-17}$	1.96 $\times \;10^{-5}$
	GBD	6.79 $\times \;10^{-9}$	0.005
2.95 m	MEM	2.34 $\times \;10^{-16}$	3.57 $\times \;10^{-5}$
	GBD	4.74 $\times \;10^{-12}$	2.63 $\times \;10^{-4}$
295 m	MEM	1.11 $\times \;10^{-14}$	0.465
	GBD	4.75 $\times \;10^{-12}$	0.629

**Table 10:** Parameter list for circular Gaussian beam clipped by a circular aperture centered in the beam waist.

Parameter	Description	Value
λ	wavelength	1064 nm
$P_{0}$	beam power	1 W
$w_{0}$	beam waist	2 mm
$z_{0}$	distance from the waist	0 mm
$R_{a}$	aperture radius	0.5 mm
*N*	mode order of the MEM	50
$w_{0d}$	waist of the modes used in the MEM	0.1 mm
*G*	grid size of the GBD	400 × 400
*L*	window size of the GBD	1.5 mm
$f_{\mathrm{ws}}$	waist scaling factor of the GBD	3/2
$w_{0g}$	grid beam waist of the GBD	0.0029 mm
grid shape	grid shape of the GBD	square
*d*	propagation distance	5 mm, 100 mm, 1000 mm, 3 Gm
*X*	number of sampling points	3001

**Table 11:** The DNMSE and the summed relative error of the MEM and GBD, respectively, for different propagation distances. The number of sampling points is 3001.

Propagation Distance	Method	Discretized NMSE $\varepsilon_{\circ }^{\mathrm{DNMSE}}$	The Summed Relative Error $\varepsilon_{\sum }^{\mathrm{rel}}$
5 mm	MEM	0.0057	0.247
	GBD	$9.58\times 10^{-4}$	0.132
100 mm	MEM	$1.9470\times 10^{-4}$	0.538
	GBD	$6.97\times 10^{-6}$	0.086
1000 mm	MEM	$2.57\times 10^{-5}$	1.68
	GBD	$6.68\times 10^{-7}$	0.328
3 Gm (2 times spot size)	MEM	$5.58\times 10^{-6}$	8.77 $\times \;10^{17}$
	GBD	$7.50\times 10^{-8}$	3.06 $\times \;10^{17}$
3 Gm ( 400 m)	MEM	$7.22\times 10^{-13}$	4.34 $\times \;10^{9}$
	GBD	$2.43\times 10^{-15}$	2.52 $\times \;10^{8}$

**Table 12:** Parameter list for the wavefront aberration.

Parameter	Description	Value
λ	wavelength	1064 nm
$P_{0}$	beam power	1 W
$w_{0}$	beam waist	1 mm
$z_{0}$	distance from the waist	0
$R_{a}$	aperture radius	1 mm
*N*	mode order of the MEM	50
$w_{0d}$	waist of the modes used in the MEM	0.2 mm
*G*	grid size of the GBD	150×150
*L*	window size of the GBD	3 mm
$f_{\mathrm{ws}}$	waist scaling factor of the GBD	8/3
$w_{0g}$	grid beam waist of the GBD	0.0267 mm
grid shape	grid shape of the GBD	square
$c_{n}^{m}$	coefficient of Zernike polynomials	10
*d*	propagation distance	5 km
*X*	number of sampling points	201×201 = 40,401

**Table 13:** Parameter list for reflection from optical components. Like in the comparison for the non-clipped Gaussian beam, we define the aperture only for implementation reasons. However, the beam is effectively not being clipped. Therefore, the complex electric fields of the MEM and GBD in the observation plane can be directly compared to the results for a fundamental Gaussian beam.

Parameter	Description	Value
λ	wavelength	1064 nm
$P_{0}$	beam power	1 W
$w_{0}$	beam waist	1 mm
$z_{0}$	distance from the waist	0
$R_{a}$	aperture radius	4 mm
*N*	mode order of the MEM	50
$w_{0d}$	waist of the modes used in the MEM	0.8 mm
*G*	grid size of the GBD	400 × 400
*L*	window size of the GBD	8 mm
$f_{\mathrm{ws}}$	waist scaling factor of the GBD	10/3
$w_{0g}$	waist of the grid beam used in the GBD	0.0333 mm
grid shape	grid shape of the GBD	square
*C*	curvatures of the mirror	0 mm${}^{-1}$, −0.002 mm${}^{-1}$, −0.02 mm${}^{-1}$, −0.1 mm${}^{-1}$
size	the diameter of the mirror	1 cm × 1 cm
*X*	number of sampling points	3001

## Data Availability

Not applicable.
